# Microenvironmental Hypoxia Induces Dynamic Changes in Lung Cancer Synthesis and Secretion of Extracellular Vesicles

**DOI:** 10.3390/cancers12102917

**Published:** 2020-10-11

**Authors:** Shun Wilford Tse, Chee Fan Tan, Jung Eun Park, JebaMercy Gnanasekaran, Nikhil Gupta, Jee Keem Low, Kheng Wei Yeoh, Wee Joo Chng, Chor Yong Tay, Neil E. McCarthy, Sai Kiang Lim, Siu Kwan Sze

**Affiliations:** 1 School of Biological Sciences, Nanyang Technological University, Singapore 637551, Singapore; swtse@ntu.edu.sg (S.W.T.); ctan088@e.ntu.edu.sg (C.F.T.); JEPark@ntu.edu.sg (J.E.P.); jeba.g@ntu.edu.sg (J.G.); NIKHIL005@e.ntu.edu.sg (N.G.); 2 NTU Institute for Health Technologies, Interdisciplinary Graduate School, Nanyang Technological University, Singapore 637553, Singapore; 3 Department of Surgery, Tan Tock Seng Hospital, Singapore 308433, Singapore; jee_keem_low@ttsh.com.sg; 4 Department of Radiation Oncology, National Cancer Centre Singapore, Singapore 169610, Singapore; yeoh.kheng.wei@singhealth.com.sg; 5 Department of Hematology-Oncology, National University Cancer Institute, National University Health System, Singapore 119228, Singapore; mdccwj@nus.edu.sg; 6 School of Materials Science and Engineering, Nanyang Technological University, Singapore 639798, Singapore; cytay@ntu.edu.sg; 7 Centre for Immunobiology, The Blizard Institute, Bart’s and The London School of Medicine and Dentistry, Queen Mary University of London, London E1 2AT, UK; n.e.mccarthy@qmul.ac.uk; 8 Institute of Medical Biology, Singapore 138648, Singapore; saikiang.lim@imb.a-star.edu.sg

**Keywords:** extracellular vesicles, hypoxia, tumour microenvironment, epithelial–mesenchymal transition, tumorigenesis, pulsed-SILAC, quantitative proteomics

## Abstract

**Simple Summary:**

Human cells can communicate with each other by releasing small packets of protein called ‘vesicles’ that are absorbed by other cells nearby and at distant locations, leading to major changes in biological activity. This is also the case for cancer cells within developing tumours, which become starved of oxygen when they outgrow local blood supplies. We wondered whether oxygen starvation might cause cancer cells to alter the protein cargo they package into vesicles, which could play an important role in supporting further tumour development. We found that under oxygen-starved conditions, cancer cells release far higher numbers of vesicles that are actively packed with proteins known to enhance tumour survival, support distribution to other body sites, and suppress patient immune responses. Cancer vesicles could therefore be used to develop new diagnostic tests that inform doctors about disease progression, and may also represent useful drug targets for new types of patient treatment.

**Abstract:**

Extracellular vesicles (EVs) mediate critical intercellular communication within healthy tissues, but are also exploited by tumour cells to promote angiogenesis, metastasis, and host immunosuppression under hypoxic stress. We hypothesize that hypoxic tumours synthesize hypoxia-sensitive proteins for packing into EVs to modulate their microenvironment for cancer progression. In the current report, we employed a heavy isotope pulse/trace quantitative proteomic approach to study hypoxia sensitive proteins in tumour-derived EVs protein. The results revealed that hypoxia stimulated cells to synthesize EVs proteins involved in enhancing tumour cell proliferation (NRSN2, WISP2, SPRX1, LCK), metastasis (GOLM1, STC1, MGAT5B), stemness (STC1, TMEM59), angiogenesis (ANGPTL4), and suppressing host immunity (CD70). In addition, functional clustering analyses revealed that tumour hypoxia was strongly associated with rapid synthesis and EV loading of lysosome-related hydrolases and membrane-trafficking proteins to enhance EVs secretion. Moreover, lung cancer-derived EVs were also enriched in signalling molecules capable of inducing epithelial-mesenchymal transition in recipient cancer cells to promote their migration and invasion. Together, these data indicate that lung-cancer-derived EVs can act as paracrine/autocrine mediators of tumorigenesis and metastasis in hypoxic microenvironments. Tumour EVs may, therefore, offer novel opportunities for useful biomarkers discovery and therapeutic targeting of different cancer types and at different stages according to microenvironmental conditions.

## 1. Introduction

Oxygen deprivation or ‘hypoxia’ is a characteristic environmental stress experienced by cancer cells within solid tumours. In order to adapt to low-oxygen conditions, tumour cells activate the hypoxia-inducible factor (HIF) pathway which drives the expression of genes that promote angiogenesis, support cell growth and proliferation, and enhance migration and tissue invasion [1]. Cancer cell adaptation to hypoxia stress is therefore associated with a more aggressive phenotype as well as increased resistance to treatment with chemotherapy and radiotherapy [2]. A better understanding of tumour cell responses to hypoxia stress will therefore be essential to developing more effective therapies to overcome tumour progression and treatment resistance.

Our lab has previously demonstrated that hypoxia stimulates cancer cell secretion of extracellular vesicles (EVs) that could potentially alter the tissue microenvironment and thereby enhance malignancy [3]. A range of biogenesis pathways can be employed to generate these EVs which vary substantially in size and are actively secreted by most cell types in the human body. EVs can be broadly classified into apoptotic bodies (50–5000 nm), ectosome/shredding microvesicles (100–1000 nm) and exosomes produced via fusion of late endosome/multivesicular bodies with the plasma membrane (30–150 nm) [4]. Exosomes in particular have been shown to play major roles in intercellular communication by the direct transfer of functionally active biomolecules including proteins and nucleic acids into the cytosol of recipient cells [5]. More recently, investigators have begun to appreciate that EVs also play a crucial role in the development of cancer via the induction of angiogenesis, ability to suppress host immunity [3,6,7], and promotion of epithelial–mesenchymal transition (EMT) in otherwise healthy cell types [8]. EMT describes the process by which epithelial cells decrease cell–cell adhesion and lose apical–basal polarity, while simultaneously upregulating mesenchymal cytoskeletal and extracellular matrix (ECM) proteins [9], which is a critical process in normal tissue development. However, cancer cells can hijack this EMT programme in order to increase motility, facilitate local invasion of healthy tissues, and undergo metastasis to remote organs. EMT can be triggered by multiple different signals including transforming growth factor-beta (TGF-β) [9], fibroblast growth factor (FGF) [10], and hepatic growth factor (HGF) [11], as well as Wnt [12] and notch family proteins [13]. These mediators ultimately modulate the expression of EMT-related genes in target cells via the induction of transcription factors including Snail, Twist, and ZEB family members [14]. While these mechanisms are now well established, it remains unknown how tumour hypoxia affects the dynamics of protein synthesis and loading into EVs that may promote disease progression. Profiling actively synthesized proteins in tumour-derived EVs could, therefore, reveal key molecular events governing the progression of hypoxic solid cancers and may lead to the identification of novel therapeutic targets for combatting these diseases.

In the current report, we used a pSILAC-based heavy isotope pulse/trace quantitative proteomics strategy to study the impact of hypoxia stress on the production of hypoxia-sensitive proteins in small EVs (<200 nm) by A549 lung adenocarcinoma cells. Our analyses of protein synthesis dynamics and EV loading under low-oxygen conditions strongly indicate that cancer EV protein synthesis is potently modulated by hypoxia stress. We also identified a group of hypoxia-sensitive EV proteins (HSEPs) that may play a critical role in stimulating EMT and metastasis within the local microenvironment. These data suggest that therapeutic targeting of vesicle-mediated signalling could offer novel strategies to complement the treatment of oxygen-deprived tumours.

## 2. Materials and Methods

### 2.1. Cell Culture

Human A549 lung adenocarcinoma cells and H1299 non-small cell lung carcinoma cells were grown in DMEM supplemented with 10% fetal bovine serum (FBS), 100 μg/mL penicillin and 100 μg/mL streptomycin at 37 °C with 5% CO_2_ in a humidified atmosphere. Hypoxic condition was achieved by purging the cell cultures with a constant flow of 95% Nitrogen gas (5% CO_2_) as monitored by a flow meter for 10 min inside a hypoxia chamber (sealed using O-ring and clamps to maintain a stable airtight environment) followed by incubation at 37 °C for a variable duration, as indicated in the text. Presence of oxygen was checked by Mitsubishi RT Anaero-Indicator (Thermo Fisher Scientific, Waltham, MA, USA).

### 2.2. Heavy Isotope Pulse/Trace for Hypoxia-Sensitive EV Proteins

Two batches of A549 cells were first grown in normoxic cultures in 100 mm dishes containing 10% FBS-DMEM that had normal “light” L-Lysine-^12^C_6_,^14^N_2_ (146 mg/L) and L-Arginine-^12^C_6_ (84 mg/L) until reaching 50% confluency in normoxia condition, at which point the medium was removed and the cells were washed twice in PBS before adding 10 mL serum-free SILAC-DMEM (Arginine/Lysine free) high glucose medium, supplemented with “heavy” L-Lysine-^13^C_6_,^15^N_2_ (146 mg/L) and L-Arginine-^13^C_6_ (84 mg/L). Then, the pulsed SILAC experiments were followed in either normoxia or hypoxia conditions in the two batches for tracing newly synthesized hypoxia sensitive EV proteins. The newly synthesized proteins labelled with heavy K/R were compared with the existing proteins with light K/R in both conditions to determine the differential protein translations in the two conditions. After 24 h incubation, the conditioned media were collected and the EVs isolated while the originating cells were retained for later protein extraction. In total, 250 mL of conditioned media were collected from 25 plates of 100 mm dishes (approximate cell count: 25–35 million cells) for each condition. The tumour-derived EVs were isolated and purified from the conditioned media as previously described for pSILAC quantitative proteomic analysis and functional assays [3,7,15]. Briefly, the conditioned media were centrifuged at 1000× *g* for 10 min to remove dead cells and debris. Supernatants were then passed through 0.22 µm filters and spin concentrated using a 100 kDa MWCO (Millipore, Sigma-Aldrich, Singapore). The supernatants were next washed with 1× PBS to remove residual media and centrifuged at 16,000× *g* for 30 min to remove large particles. The supernatants containing concentrated EVs were then diluted in 1× PBS and centrifuged at 100,000× *g* for 16 hours at 4 °C. The pellets of EVs obtained were finally re-suspended in 1× PBS and stored at 4 °C or −20 °C for immediate use or storage, respectively.

### 2.3. Physical Characterisation of EVs

The size distribution and particle quantification of the EVs sample were determined with the NanoSight NS300^®^ (Malvern Panalytical, Malvern, UK) equipped with a 488 nm blue laser and a sCMOS camera. The EVs were diluted 500 or 1000-fold in PBS prior to analysis. The analysis was performed using default protocol according to manufacturer’s software (NanoSight NS300 User Manual, Malvern, UK) and all measurement were based on three biological replicates. The parameters for the video capture were set as follows: camera level 7, slider shutter 250, slider gain 250, FPS 32.5, temperature ~24 °C, viscosity 0.906–0.910 cP, syringe pump speed 100 and capture time 60 s.

### 2.4. Proteomic Profiling of A549 EVs and Cell Lysate by pSILAC

The pSILAC proteomics analyses were conducted on A549 human lung adenocarcinoma cells and their derivative EVs generated under either normoxic or hypoxic conditions. A total of 250 µg of proteins from each sample was used for mass spectrometry analysis. The EVs were heated at 60 °C for 15 min in 100 mM TrisHCl buffer containing 4% SDS to facilitate vesicle rupture and protein release. The EV protein extracts were then mixed with an SDS-PAGE sample loading buffer and boiled at 95 °C for 10 min followed by SDS-PAGE on 12% polyacrylamide gels. Each lane was cut into five equal portions followed by further slicing into small pieces for proteomics’ profiling. Briefly, after having extensively washed the gel with 25 mM ammonium bicarbonate (ABB) buffer, a reduction in proteins was achieved by adding 10 mM Dithiothreitol (DTT) in 100 mM ABB buffer and incubating at 60 °C for 30 min. The reduced samples were then alkylated with 55 mM Iodoacetamide (IAA) in 100 mM ABB buffer at room temperature for 1 h in the dark. After washing off excess reducing/alkylating reagents, the proteins were digested using sequencing-grade modified trypsin (Promega Corporation, Madison, WI, USA) added at a ratio of 1:30 in 100 mM ABB buffer prior to overnight incubation at 37 °C. The resultant tryptic peptides were then extracted and subjected to clean-up and desalting using Sep-Pak Vac C-18 cartridge columns (Waters, Milford, MA, USA). In parallel, A549 cell were lysed with 8 M Urea in 100 mM ABB, supplemented with protease inhibitor cocktail. The protein lysate was reduced in DTT, alkylated with IAA and digested with trypsin as described (except that the reduction process was performed at 37 °C). The peptides were then desalted and fractionated with the Prominence^TM^ HPLC system (Shimadzu, Kyoto, Japan), using the XBridge^TM^ BEH C18 column (130 Å pore size. 4.6 × 250 mm, 5 µm particle size). The fractionated samples were dried and stored in −20 °C prior to LC-MS/MS analysis. A nanoHPLC instrument (Dionex, Thermo Scientific; San Jose, CA, USA) was used to perform replicate peptide injections (2 for cell lysates; 3 for EVs) into a Q Exactive device with EASY nanospray source (Thermo Fisher) at an electrospray potential of 1.5 kV. A full MS scan (350–1600 m/z range) was acquired at a resolution of 70,000 and a maximum ion accumulation time of 100 ms. Dynamic exclusion was set as 30 s. The resolution of the higher energy collisional dissociation (HCD) spectra was set to 35,000. The automatic gain control (AGC) settings of the full MS scan and the MS2 scan were 3 E6 and 2 E5, respectively. The 10 most intense ions above the 5000 count threshold were selected for fragmentation in HCD, with a maximum ion accumulation time of 120 ms. An isolation width of 2 was used for MS2. Single and unassigned charged ions were excluded from MS/MS. For HCD, the normalized collision energy was set to 28%. The under fill ratio was defined as 0.3%.

Raw data files generated from the LC-MS/MS experiments were processed using Protein Discoverer^TM^ (version 2.1.1.21, Thermo Scientific, San Jose, CA, USA) with Sequest HT and Mascot search engines employing default parameters for the QExactive Orbitrap mass spectrometer. For protein identification, Mascot and Sequest HT were used in parallel against a sequence file from the Uniprot human database (downloaded on 06 Feb 2017, 1,586,248 sequences, 61,972,042 residues). For the searches using both engines, maximum missed cleavage sites per protein was set at 2, with precursor and fragment ions mass tolerance set at 10 ppm and 0.02 Da, respectively. Carbamidomethylation (C) was set as a fixed/static modification. SILAC_R6 (R)/13C(6), SILAC_K8 (K)/13C(6)15N(2), acetylation (Protein N-term), deamidation (NQ) and Oxidation (M) were set as dynamic modifications in both search engines.

For protein identification, grouping and quantification, a consensus workflow was selected that used default settings to filter for high-confidence peptides with enhanced peptide and protein annotations. A percolator was used to calculate the target false discovery rate (FDR), which was set at 0.05 and 0.01 for relaxed and strict validation, respectively, for identified peptides. For peptide filtering, minimum peptide length was set at 6, while peptide confidence was set at ‘high’. One unique peptide sequence identified with high confidence was allowed for protein filtering. After protein grouping, the q-values for each protein group were evaluated based on target FDR. Proteins with q-value < 0.01 were designated as ‘high confidence’. Quantification of identified proteins is presented as ‘heavy isotope over light isotope’ (H/L ratio).

### 2.5. A549 EVs Treatment on Lung Cancer Cells

A549 or H1299 cells were seeded into 6-well plates (5 × 10^5^ cells/well) and cultured until 80% confluence was obtained. The cells were washed in PBS twice to remove cellular debris, followed by treatment with 50 µg of A549 EVs, generated from either normoxic or hypoxic A549 cells, in 2 mL of serum-free DMEM. For negative control, the cells were incubated with PBS-only control. After 24 h, the cells were harvested for protein and gene expression studies.

### 2.6. Western Blots

Cargo proteins were extracted from EVs by heating the vesicles at 60 °C for 15 min in 100 mM TrisHCl buffer containing 4% SDS. Proteins released from the ruptured vesicles were then mixed with gel loading buffer and boiled at 95 °C for 10 min. Protein samples were separated by SDS-PAGE on 12% polyacrylamide gels and then transferred onto nitrocellulose membranes at 100 V for 1 h. The membranes were probed with primary antibodies at 4 °C overnight. The antibodies used for Western blots were: anti-Alix antibody (#2171), anti-flotillin-1 antibody (#18634), anti-CD9 antibody (#13174), anti-caveolin-1 antibody (#3267S) from Cell Signaling Technologies (Danvers, MA, USA). The anti-hepatocyte growth factor receptor antibody (ab59884) and anti-FTH1 (ab75973) antibodies were from Abcam (Cambridge, UK). Anti-E-cadherin (sc-21791), anti-N-cadherin (sc-59987), anti-cathepsin B (sc-365558), anti-cathepsin D (sc-377299) and anti-IGFBP3 (sc-9028) antibodies were from Santa Cruz Biotechnology (Dallas, TX, USA). Anti-PLOD2 (408105) antibody was obtained from Thermo Fisher Scientific. Protein–antibody conjugates were visualized using a chemiluminescence detection kit (Thermo Fisher Scientific).

### 2.7. Total RNA Extraction and Real-Time Quantitative PCR

Total RNA content of EVs-treated A549 and H1299 cells was extracted using Nucleospin RNA kit (MACHEREY-NAGEL GmbH & Co. KG, Düren, Germany) according to the manufacturer’s protocol. Complementary DNA (cDNA) was generated from 1 µg of total RNA dissolved in DEPC-treated water using recombinant RevertAid™ M-MuLV reverse transcriptase (Thermo Fisher Scientific) for 1 h at 45 °C in a thermal cycler (Bio-Rad, Hercules, CA, USA) and used for real-time quantitative PCR (RT-qPCR) experiments. Briefly, cDNA was mixed with 2X KAPA SYBR^®^ FAST qPCR Master Mix (Thermo Fisher Scientific) and gene-specific primers and subjected to the following conditions: denaturation at 95 °C for 15 s followed by annealing at 60 °C for 15 s and extension at 72 °C for 15 s then final extension at 72 °C for 10 min over a total of 40 cycles. Gene expression was measured in three biological replicates, using 60S acidic ribosomal protein P0 (RPLP0) as the housekeeping gene. The primer sequences of target genes were as follows: Snail forward: 5′-TTTACCTTCCAGCAGCCCTA-3′ Snail reverse: 5′-CCCACTGTCCTCATCTGACA-3′; Slug forward: 5′-TGTTGCAGTGAGGGCAAGAA-3′; Slug reverse: 5′-GACCCTGGTTGCTTCAAGGA-3′; RPLP0 forward: 5′-TCGACAATGGCAGCATCTAC-3′; RPLP0 reverse: 5′-GCCTTGACCTTTTCAGCAAG-3′; VIM forward: 5′-GAGAACTTTGCCGTTGAAGC-3′; VIM reverse: 5′-TCCAGCAGCTTCCTGTAGGT-3′; COL1A1 forward: 5′-GTGCTAAAGGTGCCAATGGT-3′; COL1A1 reverse: 5′-CTCCTCGCTTTCCTTCCTCT’; COL4A1 forward: 5′-GAAGGGTGATCCAGGTGAGA-3′; COL4A1 reverse: 5′-CACCCTTGTCACCTTTTGGT-3′; COL5A1 forward: 5′-GTGGCACAGAATTGCTCTCA-3′; COL5A1 reverse: 5′-TCACCCTCAAACACCTCCTC-3′; COL18A1 forward: 5′-GGGACCTGTGGTCTACGTGT-3′; COL18A1 reverse: 5′-CTCTCCCTTGGCTCCTTTCT-3′; E-Cadherin forward: 5′-TGGAGGAATTCTTGCTTTGC-3′; E-Cadherin Reverse: 5′-CGTACATGTCAGCCAGCTTC-3′; N-Cadherin forward: 5′-TGCAAGACTGGATTTCCTGA-3′; N-Cadherin Reverse: 5′-CTCTGCAGTGAGAGGGAAGC.

### 2.8. Fluorescence Microscopy

A total of 1 × 10^5^ A549 or H1299 cells/well were seeded onto cover slips in a 6-well plate and cultured overnight to ensure cell attachment. Cells were then washed with PBS and incubated in serum-free DMEM containing 50 µg of ‘normoxic A549 EVs’ or ‘hypoxic A549 EVs’ (or PBS-only control). After 24 h incubation, the cover slips were washed twice with PBS, fixed with 4% paraformaldehyde solution for 15 min, and washed three times in PBS. After cell fixation, the cells were permeabilized using 0.1% triton X-100 in PBS for 10 min followed by three washes in PBS. Cells were blocked with 5% BSA in PBS for 30 min at room temperature and then incubated with Alexa Fluor^®^ 488 Phalloidin (1:200, Cell Signaling Technology, #8878, Beverly, MA, USA) in the same blocking solution for minimum 30 min at room temperature. Finally, the cover slips were washed three times with PBS followed by mounting onto glass slides and monitoring for stress fiber formation using fluorescence microscopy.

### 2.9. Wound Healing and Migration Assay

The wound healing and migration assay was adapted from the method described Bobadilla et al. [16]. Briefly, A549 or H1299 cells were seeded into 6-well plates (5 × 10^5^ cells/well) and cultured until 90% confluence. A sterile pipette tip (200 μL) was used to scratch a single straight wound along the central axis of the well. After removing cell debris by washing in PBS, cells were incubated in serum-free DMEM in the presence or absence of 50 µg A549-derived EVs generated under either normoxic or hypoxic conditions (or PBS-only control). Photographs were taken at baseline and 24 h using a bright field microscope at 4× magnification. A gap at 0 h was considered as 100% and cell migration was evaluated as % closure of the wound area after 24 h culture. The average area of the gaps was calculated using Image J software (NIH, Bethesda, ME, USA).

### 2.10. Invasion Assay

The invasion assay was performed in transwell assay chambers with 8-μm pore size (Corning Inc., Corning, NY, USA) and ECM (Sigma Aldrich, Singapore) coating as previously described [17]. A549 and H1299 cells were added separately at a density of 5 × 10^4^ cells in the upper chamber of each transwell together with 20 µg normoxia or hypoxia-derived A549 EVs in serum-free DMEM. The lower chamber contained 10% FBS-DMEM. After 24 h, cells that remained in the upper chamber were removed with cotton swab soaked in PBS. Cells that invaded into the lower chamber were fixed in 100% methanol and then stained with 0.1% crystal violet for cell counting with a bright field microscope at 10× magnification.

### 2.11. Transmission Electron Microscopy

A549-derived EVs were 20-fold diluted in PBS and a 7 µL volume added onto a glow discharged carbon-coated grid and incubated for 1 min. Thereafter, 2% uranyl acetate was added to the sample and incubated for 1 min. Excess uranyl acetate was blotted off with filter paper. The grid was air-dried for 10 min and subsequently imaged using the T12 Icorr transmission electron microscopy (TEM) at 120 kV (FEI Company, Hillsboro, OR, USA).

### 2.12. Statistical Analysis

Results presented as dot plots and bar plots were generated from Graphpad Prism 5.0 (Graphpad Software, San Diego, CA, USA), using the mean +/− standard error of mean (S.E.M) and discrete variable, respectively. Statistical comparisons were performed using unpaired T-tests in Graphpad software. *p*-values < 0.05 were considered significant.

## 3. Results

### 3.1. Oxygen Deprivation Promotes EV Secretion by A549 Human Lung Cancer Cells

**Figure 1:**
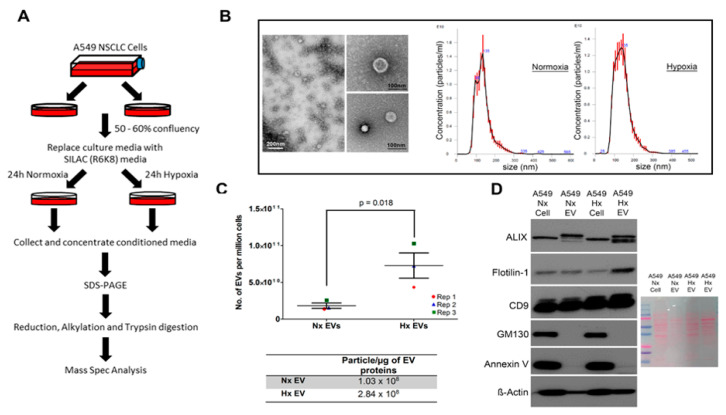
Microenvironmental hypoxia promotes extracellular vesicles (EVs) release by A549 human lung cancer cells. Schematic of the pSILAC experimental workflow. Standard culture medium was replaced with serum-free SILAC medium (R6K8) immediately prior to cancer cell incubation in Normoxic (Nx) or Hypoxic (Hx) conditions for 24 h. Extracellular vesicles (EVs) released during this period were then isolated and concentrated for subsequent analysis by tandem mass spectrometry (**A**). Physical characteristics of the vesicles isolated from cancer cell cultures as assessed by transmission electron microscopy and Nanosight light scattering approach. Scale bar is 200 nm and 100 nm for large and small TEM images respectively. (**B**). Nanosight quantification of EVs output derived from non-SILAC labelled A549 cells in either normoxic or hypoxic conditions for 24 h. Error bars indicate S.E.M of three biological replicates. Number of particles secreted in each condition were normalized against EVs protein concentration (**C**). Western blots (left) of archetypal exosome markers (ALIX, Flotilin-1, CD9) in the EVs isolated from normoxic and hypoxic tumour cell cultures. Absence of GM130 (golgi) and Annexin V (apoptotic body) expression in the EVs sample indicate absence of cellular contamination. Ponceau S staining as protein loading control (right) (**D**). Detailed information about western blot can be found at Figure S1.

EVs are released by hypoxic tumour cells into the local microenvironment to modulate its survival but the effect of tumour hypoxia on the dynamics of protein synthesis and loading into EVs remains unclear. We therefore sought to test the hypothesis that hypoxia may trigger de novo synthesis of novel proteins which may be selectively incorporated or loaded onto EVs alongside pre-existing cellular components. To do this, we used a pSILAC-based proteomic approach to analyse the protein cargo of EVs produced by A549 lung cancer cells when deprived of oxygen. Cells were first grown to 50% confluence under standard culture conditions and then supplemented with C13-labelled heavy lysine and arginine (which are rapidly incorporated into newly synthesized proteins) before continuing culturing the cells in normoxic or hypoxic environment for 24 h (Figure 1A). We then isolated tumour-derived vesicles by ultracentrifugation and used tandem mass spectrometry to assess the dynamics of EVs loading with pre-existing cellular proteins (containing light K and R amino acids) versus newly synthesized proteins (labelled with heavy K and R) during the culture period. A total of 250 µg of EVs-derived proteins were required for mass spectrometry analysis, equivalent to the EV output of approximately 30 million cells for each condition. The vesicles obtained with this method displayed physical features characteristic of exosomes such as cup-shaped morphology from electron microscopy imaging, had a mean particle size 138–142 nm diameter (range 30–150 nm) and EV dimensions were not significantly different between EVs from hypoxic cultures and normoxic cultures (Figure 1B). While the physical properties of EVs were highly consistent between culture conditions, oxygen deprivation induced a significant increase in the absolute quantity of particles released by lung cancer cells (Figure 1C), thus confirming that hypoxia can exert potent effects on tumour EVs production. Lastly, Western blotting confirmed that both conditions produced vesicles containing archetypal protein markers of exosomes including ALIX, Flot1 and CD9 and were not contaminated with cellular organelles such as golgi apparatus (GM130) or apoptotic body (Annexin V) (Figure 1D).

### 3.2. Hypoxia Stress in Lung Cancer Cells Induces De Novo Synthesis of Specific EV Proteins

**Figure 2:**
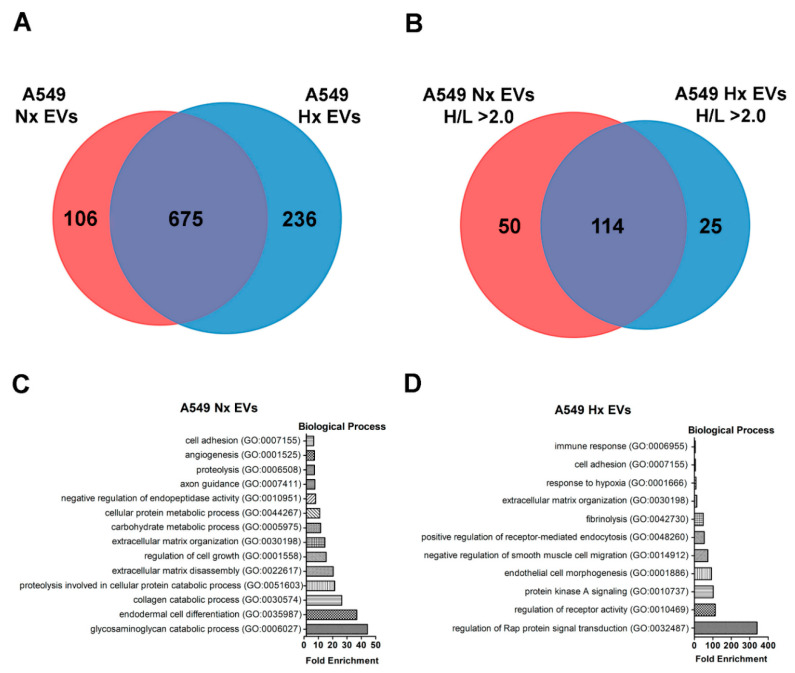
Selected EVs proteins are rapidly synthesized by A549 lung cancer cells during hypoxia stress. Venn diagram of EV proteins specific to normoxic cultures, unique to hypoxic cultures, or dispersed under both conditions (**A**). Venn diagram showing the proportion of rapidly/recently synthesized EVs proteins (H/L ratio ≥ 2) detected in the normoxia dataset, hypoxia dataset, or shared by both (**B**). Gene ontology analysis revealed biological processes associated with newly synthesized EVs proteins that are found exclusively in normoxic condition and Nx EVs proteins that have a higher synthesis rate than its Hx EVs counterpart ($\frac{Nx\;\frac{H}{L}ratio}{Hx\;\frac{H}{L}ratio}$ ≥ 1.5) (**C**) as compared to newly synthesized EVs proteins that are found exclusively in hypoxic condition and Hx EVs proteins that have a higher synthesis rate than its Nx EVs counterpart ($\frac{Hx\;\frac{H}{L}ratio\;}{Nx\;\frac{H}{L}ratio}$ ≥ 1.5) (**D**).

We next assessed the protein cargo of tumour-derived EVs released into normoxic and hypoxic cultures, as well as the vesicle content of newly synthesized proteins. Using pSILAC-based quantitative proteomics, we achieved high-confidence identification of ~900 proteins in EVs derived from normoxic lung cancer cells, and 1046 proteins in EVs released by their hypoxic counterparts. The top 25 EV markers compiled in ExoCarta database [18] and Vesiclepedia [19] were identified (Tables S1 and S2). After initial data sorting to remove proteins that lacked a measurable heavy-to-light (H/L) ratio, we confirmed that 675 proteins were shared between the two datasets, suggesting that these molecules are common components of EVs produced by A549 lung cancer cells irrespective of culture conditions. A further 106 proteins were detected only in normoxic cancer-derived EVs (Nx EVs), and 236 proteins were present exclusively in hypoxic cancer-derived EVs (Hx EVs), suggesting that the cellular expression and vesicle loading of these molecules depends on microenvironmental oxygen availability (Figure 2A; File S1).

Using a H/L isotope ratio ≥ 2 as a cut-off value for identifying newly synthesized molecules, we observed that hypoxia was associated with the active production and EV loading of 139 distinct proteins, compared with 164 proteins under standard culture conditions (File S1. Among these EVs proteins, 25 of them were more rapidly synthesized in a low-oxygen environment (Figure 2B), including several de novo synthesized proteins (H/L ratio = 100) (Table 1). Based on the H/L ratio and emPAI value, we found that a subset of these EV proteins (including GOLM, LAMB2, EIF4A3, and MET) were identified in both normoxic and hypoxia A549 cells but were preferentially sorted into the EVs under hypoxic condition, while the expression of other proteins (including ANGPTL4, STC1, SDC-ROS1 and IGFBP3) was induced exclusively under hypoxic conditions, as they were detected in hypoxic A549 cells and derivative vesicles only (Table 1). The expression of several key proteins were validated by Western blot (using non-SILAC EV samples), which correlated well with the results obtained by tandem mass spectrometry (Figure S2). Our data now reveal that a distinct subset of these HSEPs is synthesized de novo and preferentially loaded into the EV cargo under hypoxic conditions. Furthermore, our analysis also identified that oxygen deprivation triggers EV loading of the proteins B3GAT2 and C1orf112, whose role in human carcinogenesis is currently unknown.

Next, gene ontology analysis was performed on newly synthesized proteins that are enriched in the EVs under either normoxic or hypoxic conditions [20]. The analysis revealed that newly synthesized proteins enriched in Nx EVs are involved in biological processes associated with biomolecule metabolism such as glycosaminoglycan catabolic process (GO:0006027) collagen catabolic process (GO:0030574), carbohydrate metabolic process (GO:0005975) and cellular protein metabolic process (GO:0044267) (Figure 2C), whereas newly synthesized proteins enriched in Hx EVs partake in biological processes involved in regulation and signaling activities. This includes the regulation of Rap protein signal transduction (GO:0032487), regulation of receptor activity (figureGO:0010469), protein kinase A signaling (GO:0010737), response to hypoxia (GO:0001666) and immune response (GO:0006955) (Figure 2D). On the other hand, some of the newly synthesized proteins in both Nx EVs and Hx EVs mediated similar processes such as cell adhesion (GO:0007155) and extracellular matrix organization (GO:0030198). Furthermore, the top annotation cluster highlighted cellular component such extracellular matrix (Figures S3 and S4) that is highly enriched in both culture conditions, whereas other cluster identified biological process related to glycolipid and oligosaccharide metabolic processes were enriched in hypoxic conditions only. 

### 3.3. Altered Dynamics of Cancer EV Production and Release during Hypoxia Stress

**Figure 3:**
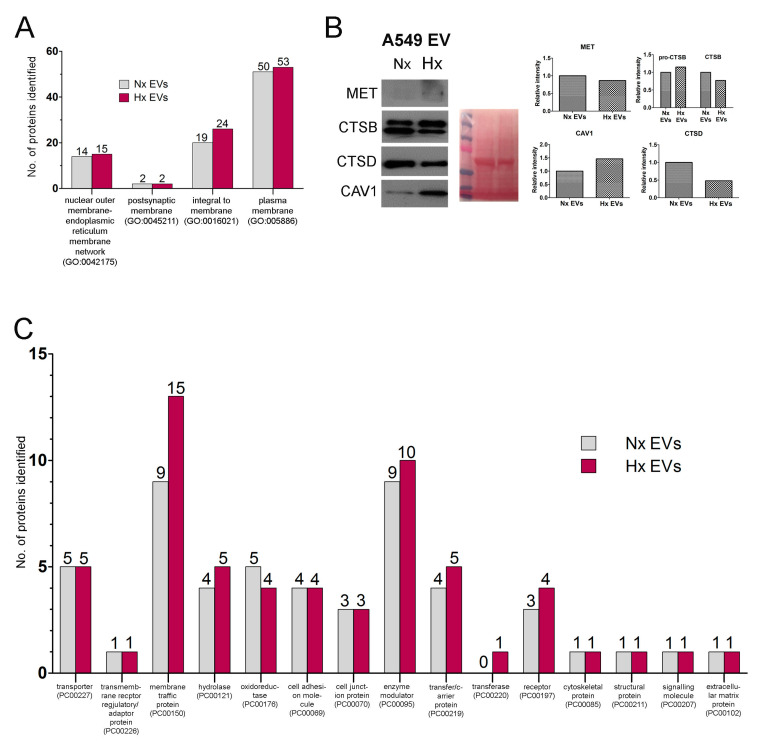
Functional classification of EVs proteins synthesized by hypoxic A549 lung cancer cells. (**A**) Bar chart showing distribution of membrane proteins according to sub-cellular localization (as assigned by the PANTHER functional classification system. (**B**) Western blots (left) confirming expression levels of selected membrane-associated proteins by A549 cells when subjected to hypoxia (Hx) or normal culture conditions (Nx). Experiment was done with 3 biological replicates. Ponceau S staining as protein loading control (center) and the analysis was quantified by densitometry (right). (**C**) Bar chart showing distribution of membrane proteins according to functional class. Detailed information about Western blot can be found at Figure S1.

To further understand how cancer EV biogenesis is modified by oxygen deprivation, we next used the PANTHER system to classify vesicle protein content based on reported subcellular localization [32], and assessed whether this was significantly modified by hypoxia stress. A549 cell-derived EVs were comprised primarily of an integral membrane and plasma membrane proteins (Figure 3A) which displayed low rates of synthesis (Table 2). This suggests that hypoxia reduces overall translational activity despite marked increases in EV secretion and active synthesis of HSEPs, which is in line with other studies [33].

Our data also suggest that during hypoxia stress, lung cancer cells either alter the distribution of existing cellular proteins to the membrane fraction of EVs or can secrete EV types with a different membrane protein configuration. For example, cathepsin B (CTSB) and Syntenin-1 (SDCBP) were abundant within the cargo of Nx EVs, but both proteins displayed a stark reduction in synthesis rate and abundance in Hx EVs (Figure 2B–D, Figure 3B). In contrast, several other proteins including caveolin 1 (CAV1), sulfhydryl oxidase 1 (QSOX1), integrin beta 1 (ITGB1), and ceruloplasmin (CP) were instead enriched in EVs released by hypoxic cells (high emPAI value; Table 2), despite their reduced rates of synthesis (as determined by H/L ratio). 

Further classification of our proteomic data revealed that hypoxia altered the EV loading of proteins related to intracellular membrane trafficking (Figure 3C, Table 3). As shown in Table 3, oxygen restriction was associated with a marked reduction in the EV abundance of syntenin 1, which critically regulates the syndecan–syntenin (SDCBP)-ALIX (PDCD6IP) intracellular vesicle formation pathway [34]. In contrast, Hx EVs appeared to be enriched in clathrin heavy chain (CLTB), clathrin light chain (CLTC), and key mediators of cargo selection and transport between the trans-golgi network (TGN), endosomes and lysosomes. For instance, hypoxia conferred 2–5-fold upregulation in EV abundance of the ‘retromer’ complex of vesicle protein sorting 35 (VPS35), VPS29, and VPS26A, which controls the reverse transport of protein cargo back to the Trans-golgi network (TGN) (Table 3). Similarly, only EVs released by hypoxic tumours were found to contain the ‘coatomer’ complex of subunits alpha (COPA), beta (COPB1), and epsilon (COPE), which mediate protein cargo transport between the Golgi and endoplasmic reticulum.

The potential survival advantage that the EVs confer to developing tumours was also underlined by our observation that hypoxia induced marked changes in the EVs loading of E-cadherin (CDH1) and N-cadherin (CDH2), which are essential protein mediators of epithelial-mesenchymal transition (EMT) in epithelial cancers. While CDH1 was readily detected in Nx EVs (with moderate H/L ratio), this protein was absent from Hx EVs. In contrast, EVs released by hypoxic cancer cells contained detectable levels of CDH2 (Table 2), which are typically not overexpressed by tumours under standard culture conditions [35]. These data indicate that hypoxia not only induces changes in EVs content of plasma membrane proteins but also triggers substantial changes in production dynamics. Indeed, since hypoxia is known to induce EMT in epithelial cancer cells via the down-regulation of E-cadherin and concomitant up-regulation of N-cadherin [36], these results strongly suggest that EVs released by hypoxic cancer cells could be a major trigger for EMT in vesicle recipients.

### 3.4. Lung Cancer-Derived EVs Promote Epithelial-Mesenchymal Transition (EMT)

**Figure 4:**
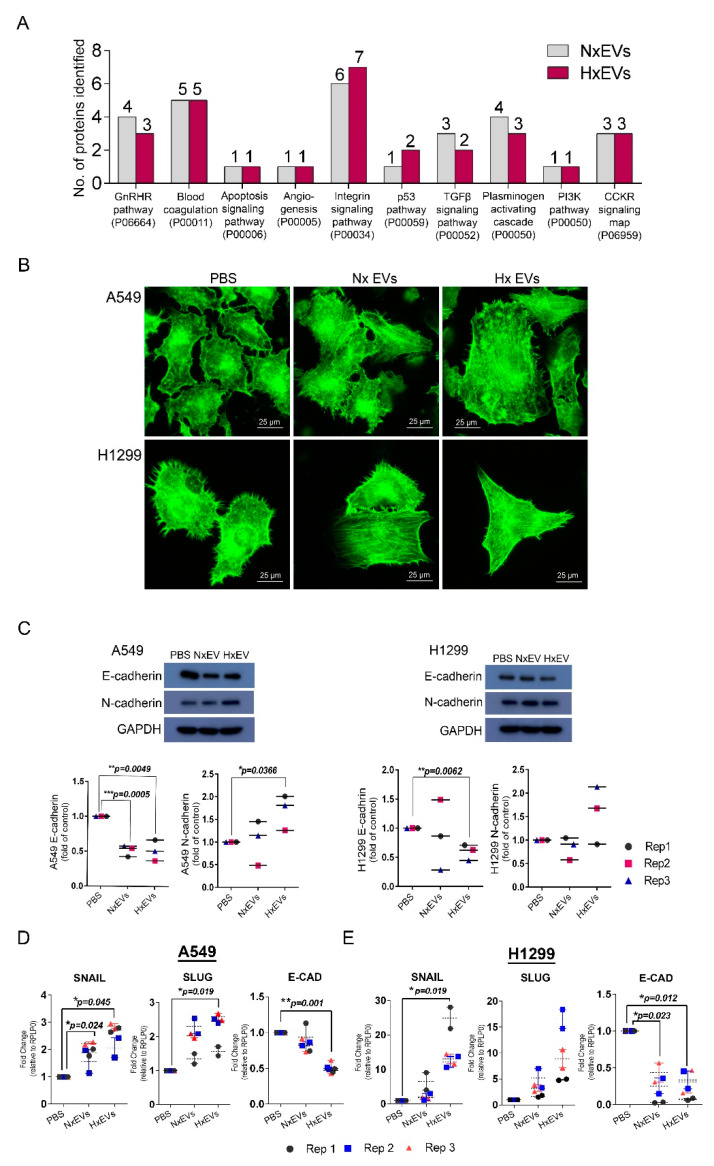
EVs released by oxygen-starved tumours promote EMT in recipient A549 lung cancer cells. Bar chart showing signaling pathways triggered by EVs derived from A549 lung cancer cells in normoxic cultures (Nx) or hypoxic conditions (Hx) based on the PANTHER functional classification system (**A**). Representative fluorescent images of EV-treated A549 lung cancer and EV-treated H1299 lung cancer showed actin rearrangement as revealed by staining with phalloidin (green). Magnification = 100× (**B**). Western blots of E-cadherin and N-cadherin expression levels in A549 cells and H1299 cells when subjected to hypoxia or normoxia derived EVs treatment; experiment was repeated with 3 biological replicates. Error bars indicate S.E.M of three biological replicates (**C**). Gene expression levels of characteristic EMT markers, Snail, slug and E-cadherin in A549 cells (**D**) and H1299 cells (**E**) after EV treatment. Error bars indicate S.E.M of three biological replicates. Detailed information about Western blot can be found at Figure S1.

**Figure 5:**
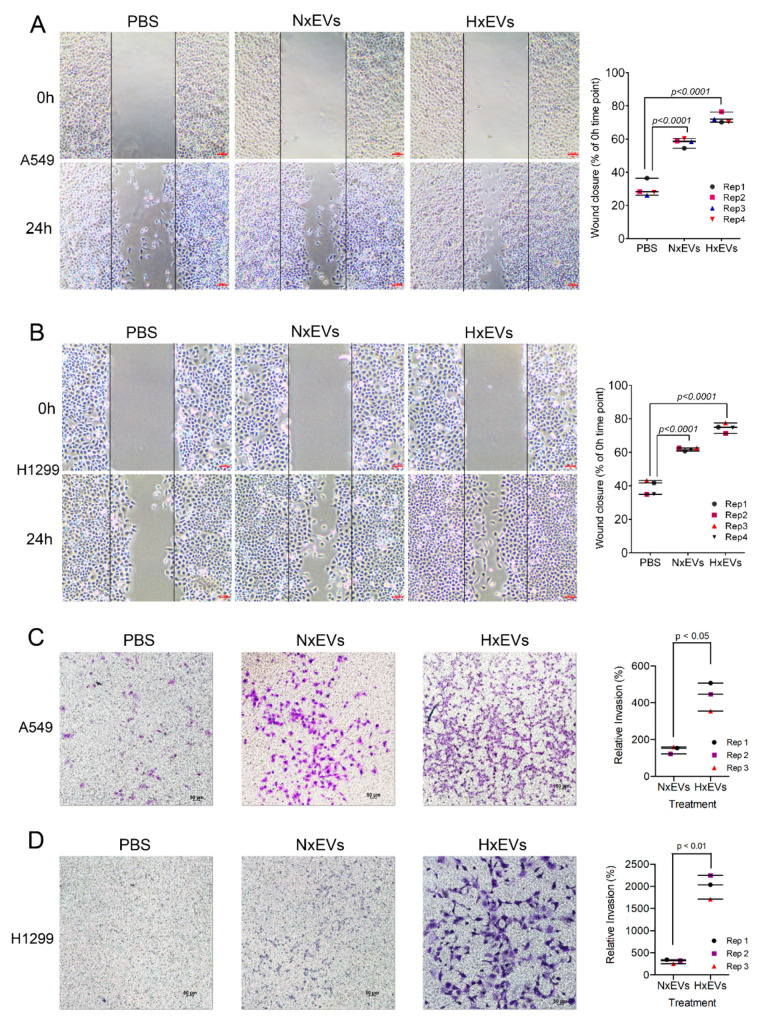
A549 EVs promote migration and invasion in recipient A549 and H1299 lung cancer cells. Representative images showing EV-induced wound closure of A549 cells (**A**) and H1299 cells (**B**) in wound healing assays conducted over 24 h. Dot plot illustrating the rate of wound closure based on percentage against time-point 0 h. Error bars indicate S.E.M of four biological replicates. Representative images showing EV-induced invasion of A549 cells (**C**) and H1299 cells (**D**) across the ECM gel after 24 h incubation. The migrated cells were counted after staining with crystal violet. Dot plot illustrating the number of cells invade and migrate the ECM gel upon Nx EVs or Hx EVs incubation according to the percentage of invasion. Error bars indicate S.E.M of three biological replicates. Scale bar = 100 µm (**A**,**B**) and 50 µm (**C**,**D**).

We investigated whether the protein cargo of the extracellular vesicles could trigger signalling pathways that promote tumorigenesis (Figure 4A). Functional cluster analysis revealed that Hx EVs were also significantly enriched in other EMT-linked signalling protein involved in TGFβ and PI3K signalling pathway. We therefore proceeded to test the functional relevance of this observation by isolating EVs from both hypoxic and normoxic A549 cells and assessed their impact on the morphology/migration of on A549 cells as well as validating the observation in another lung cancer cell line, the H1299 non-small cell lung cancer line. Based on phalloidin staining, EV treatment led to marked changes in the recipient tumour cell’s filamentous actin (F-actin) organization (Figure 4B). After 24 h of incubation with either A549 Nx EVs or Hx EVs, the typical arrangement of cortical F-actin in A549 and H1299 cells were transformed into long actin filaments, which are characteristic of EMT [37]. This observation was accompanied by a decrease in the gene and protein expression of E-cadherin in lung cancer cells treated with Hx EVs (Figure 4C–E). Hx EVs also stimulated RNA up-regulation of the E-cadherin repressors SNAI1 (Snail) and SNAI2 (slug) in A549 recipients, and increased slug gene expression in H1299 cancer cells. Intriguingly, only EVs derived from hypoxic A549 cells were capable of upregulating N-cadherin expression in A549 recipient tumour cells (Figure 4C). Cancer cells that acquire mesenchymal characteristics gain enhanced ability to migrate and invade healthy tissues. We thus performed both cell invasion and wound-healing assays to examine the migratory and invasive potentials of both A549 and H1299 lung cancer cells after EV treatments. A549 and H1299 lung cancer cells demonstrated increased migratory properties 24 hours after EVs exposure, as indicated by the higher rate of wound closure when compared to the PBS control (Figure 5A,B). A549 Hx EVs also induced a 15–20% increase in migration rate in both recipient cells in four replicates when compared to A549 Nx EVs treatment. We next determined if A549 Hx EVs treatment induced a greater invasion and migration rate across the ECM in recipient cells instead. The transwell invasion assay indicated that A549 Hx EVs treatment on both A549 and H1299 lung cancer cells significantly enhanced cell invasion across the ECM gel when compared to A549 Nx EVs (Figure 5C,D). Collectively, these data confirm that A549 Hx EVs trigger morphological and molecular changes in their recipient cells that are consistent with EMT induction events [38].

## 4. Discussion

Tumour-derived EVs are now well-recognized to enhance cancer cell adaptation to changing microenvironmental conditions including oxygen starvation, but it has remained unclear how hypoxic stress modifies protein dynamics and loading into EVs to modulate these events. Using a pSILAC-based quantitative proteomics method to pulse/trace hypoxia sensitive EV proteins, we now report that oxygen deprivation triggers rapid tumour synthesis of a specific subset of EV proteins that can induce profound changes in cancer cell morphology, migration and invasive potential. In addition to increasing rates of EV release, as has been reported previously, we observed that tumour hypoxia also induces dynamic changes in EVs/exosome biosynthetic pathways and cargo loading, which could affect tumour malignancy and potentially impact patient outcomes. Tumour EVs/exosomes may, therefore, represent useful therapeutic targets or biomarkers for particular cancer types/stages according to the microenvironmental conditions in which they are developing. EVs carry cargo that mimics the composition of their parent cell types and could thus be used as non-invasive biomarkers of cancer progression in human patients. Highly proliferative solid tumours rapidly outgrow local blood supplies, resulting in low oxygen levels in the local microenvironment and subsequent cancer cell adaptation towards a more aggressive phenotype that often includes chemo-resistant and radio-resistant properties [2]. In the current report, we identified, for the first time, that this process also entails selective expression of a specific subgroup of hypoxia-sensitive EV proteins (HSEPs) that were expressed and packaged into EV cargo under low-oxygen conditions. Through the study of protein synthesis rate and protein abundance in normoxic and hypoxic A549 cells and EVs, we were able to identify proteins that are highly synthesized and preferentially sorted into the EVs under hypoxic conditions.

Gene ontology analyses on newly synthesized proteins enriched in either Nx EVs or Hx EVs revealed different biological processes that they governed cancer progression. In particular, Hx EVs are enriched in newly synthesized proteins involved in regulation and signaling activities and highlight their ability to promote intercellular communication for cancer progression. Several of these HSEPs have been reported in other contexts to play key roles in tumour progression by cancer cell proliferation (NRSN2, WISP2, SPRX1, LCK), metastasis (GOLM1, STC1, MGAT5B), and stemness (STC1, TMEM59), as well as promoting angiogenesis (ANGPTL4) and host immunosuppression (CD70). For example, we observed the de novo synthesis and EV secretion of the multifunctional secretory protein ANGPTL4 under hypoxic conditions only. Since ANGPTL4 exerts wide-ranging effects on diverse processes including glucose and lipid metabolism [39], inflammation, and angiogenesis [40,41,42], it is possible that hypoxia-induced synthesis and dispersal of this protein could significantly alter the course of tumour development. Indeed, we also detected potent effects of hypoxia on cancer cell expression and EV loading of multiple other proteins that are likely capable of impacting on cancer progression; Neurensin 2 (NRSN2) regulates phosphorylation levels of Akt and mTOR to modulate cell viability and proliferation via the PI3K/Akt/mTOR pathway [43], secretory protein WNT1-inducible signalling pathway protein 2 (WISP2/CCN5) has been reported to exert mitogenic/survival effects in various cancer types [44,45], Sushi-Repeat-Containing Protein X Chromosome (SRPX/DRS) is a tumour suppressor gene/membrane protein known to regulate glucose metabolism [46], and tyrosine-protein kinase Lck (LCK) is a proto-oncogene aberrantly expressed in various malignancies [47]. We further identified other HSEPs that are known to promote cancer invasiveness. This included the Golgi Membrane Protein 1 (GOLM1), which is involved in intracellular protein trafficking and can upregulate matrix metalloproteinase-13 (MMP13) via CREB-mediated transcriptional activity [48,49], GOLM1 may also serve as a novel molecular target for preventing EMT in metastatic hepatocellular carcinoma [50,51]. In addition, we observed that A549 Hx EVs were enriched in the golgi-expressed glycosylation enzyme alpha 6-mannosylglycoprotein 6-beta-N-acetylglucosaminyltransferase B (MGAT5B) which is a critical regulator of cancer cell–cell contact, EMT, and metastasis [52]. Furthermore, the α-ketoglutarate-dependent enzyme PLOD2 can hydroxylate pro-collagen within the ER and has been implicated in hypoxic cancer cell invasion in various experimental models [30,53,54,55,56,57]. Taken together, these data indicate that hypoxia stress stimulates cancer cell protein synthesis and EV distribution to induce diverse functional effects that likely extend beyond local survival strategies. Indeed, our data indicate that hypoxic tumours also load EVs with proteins including stanniocalcin 1 (STC1) and transmembrane protein 59 (TMEM59/DCF1), which have been reported to induce stem-like traits in recipient cancer cells via activation of the NOTCH1-SOX2 signalling pathway and control of cellular differentiation, respectively [58,59,60,61]. It is clear, therefore, that hypoxia significantly modifies cancer EV biology to induce a wide range of pathological effects that are not yet fully understood.

In addition to conferring biological advantage to tumour cells, EV distribution of tumour proteins has also been shown to promote immune suppression of the host, with likely detrimental effects on clinical outcome. In the current report, hypoxia stress was observed to promote EV loading of the membrane-bound checkpoint protein CD70 which has been implicated in immune evasion via several different mechanisms [62,63,64]. In addition, an increase in the levels of trimeric CD70 at the outer membrane can activate MAPK/PhoE signalling and may instead enhance melanoma cell invasiveness [65].

Next, we demonstrated that the findings from our proteomics dataset correlated well with the assay performed to understand the effect of Hx EVs treatment on its recipient cells. In our assays, A549 lung-cancer-derived EVs induced epithelial-mesenchymal transition (EMT) in recipient lung cancer cells. In particular, treatment with Hx EVs exhibited dramatic changes in morphology, migration, and invasive potential. These data highlighted that tumour-derived EVs derived from hypoxic condition are likely to promote metastasis and could potentially represent novel therapeutic targets for metastatic cancers. Indeed, metastasis is a hallmark feature of cancer and major cause of death that requires multiple molecular events, including EMT, to occur in the correct sequence [66]. EVs have been suggested to act as critical mediators of intercellular communications within the tumour microenvironment [5,67,68,69], and may therefore be important for organising the molecule events that facilitate metastasis. Hypoxic EV-treated A549 cells also displayed rapid decreases in E-cadherin expression in parallel with increased N-cadherin expression, which is typical of oncogenic EMT [70]. Intriguingly, this cadherin switching event may occur readily in different types of tumour, since H1299 cells treated with Hx EVs exhibited a decrease in E-cadherin expression but did not upregulate N-cadherin. Since hypoxia is known to induce EMT in epithelial cancer cells via down-regulation of E-cadherin and concomitant up-regulation of N-cadherin [36], these results strongly suggest that EVs released by hypoxic cancer cells could be a major trigger for EMT in vesicle recipients.

In addition, we observed that Hx EVs exposure to A549 and H1299 cancer cells promoted greater wound closure when compared to the control. It also increased the speed of wound closure of cancer cells treated with Nx EVs by 15–20% in all four replicates despite the difference being not statistically significant. This may reflect the fact that 443 of the common EV proteins isolated from both conditions had similar H/L ratios. Gene ontology analysis revealed an enrichment of the Wnt signalling pathway process that is known to promote EMT (Figure S5) [71]. In this cluster, Rac Family Small GTPase 1 (RAC1), Caveolin 1 (CAV1), Low-density lipoprotein receptor-related protein 6 (LRP6) and collagen type I alpha 1 chain (COL1A1) were identified as proteins shown to promote EMT in various cancer. RAC1 signalling was shown previously to be a key mediator of cancer migration modulating F-actin organization in colorectal cancer [72], while CAV1 has been demonstrated to promote cancer growth and migration in lung cancer [73]. The overexpression of LRP6, a co-receptor of the Wnt/catenin pathway, was shown to enhance cancer formation, proliferation, and migration in liver cancer [74]. Lastly, COL1A1 was able to activate the Wnt signalling pathway via RAC1 and promote migration in colorectal cancer [75]. Therefore, it is likely that both A549 Nx EVs and Hx EVs are able to influence migratory properties in recipient cells.

On the other hand, we could not exclude the possibility that lipids, nucleotides, and metabolites, in addition to proteins in the Hx EVs cargo, can induce EMT on tumour cells. EMT-inducing microRNAs such as miR-191, let-7a and miR2-23a [8,76], and long, non-coding RNAs such as lncRNA-ATB, have been reported to release ZEB1 and ZEB2 from suppression to drive EMT in hepatocarcinoma cells [77]. Therefore, the observed EVs-induced EMT in our experiment may co-depend or synergistically depend on these biomolecules in the EVs cargo. As our study seeks to investigate the effect of hypoxia on protein synthesis and loading in EVs focusing on proteomics, the above aspects are beyond the scope of this investigation.

EVs carry numerous bioactive components that closely reflect the cell type and tissue of origin as well as the mechanism by which they are formed, which can be influenced by microenvironmental conditions such as hypoxia [67]. We and others have identified hypoxia as a major influence on EV secretion by multiple different types of cancer [3], and the current report is consistent with the findings from other investigators, who have also observed that low-oxygen stress significantly increases EV production in multiple types of tumour, including breast cancer [78], A431 squamous skin carcinoma cells, and B16 murine melanoma cells [7]. Since environmental stresses such as hypoxia are critical factors driving tumour progression and metastasis, it is likely that dynamic changes in EVs’ synthesis and release significantly alter cancer cell interactions with local tissues and host leukocytes.

## 5. Conclusions

In conclusion, hypoxia has a profound effect on cancer cells’ survival and progression, and cellular protein synthesis is heavily modulated under this condition. Cancer-cell-derived EVs are an important element for cancer cells survival as they promote intercellular communication to regulate an array of biological processes such as angiogenesis, immune regulation and cell migration and invasion. We have therefore demonstrated the usage of the pSILAC methodology in understanding protein dynamics such as protein synthesis and loading into lung cancer cells EVs in response to hypoxia. Our data indicate that A549 lung cancer cells not only secrete more EVs under hypoxic conditions, but also rapidly modify the protein cargo of these vesicles, which can promote EMT/metastasis in non-transformed recipient cells. These findings suggest that the therapeutic targeting of HSEPs in small extracellular vesicles may provide complementary treatment strategies for cancer control, particularly in situations where EVs communication plays a critical role.

## Figures and Tables

**Table 1:** EV proteins rapidly synthesized by A549 lung cancer cells in response to hypoxia.

Protein Names (Gene Symbols)	A549 Cells (Nx)		A549 EVs (Nx)		A549 Cells (Hx)		A549 EVs (Hx)		Hypoxia Inducible Gene
	H/L	emPAI	H/L	emPAI	H/L	emPAI	H/L	emPAI	
Angiopoietin-Related Protein 4 (ANGPTL4)	-	-	-	-	-	0.122	100	0.413	Yes [21]
Neurensin 2 (NRSN2)	-	-	-	-	-	-	100	0.233	-
WNT1-Inducible-Signaling Pathway Protein 2 (WISP2)	-	-	-	-	-	-	100	0.212	Yes [22]
Isoform 2 of CD70 Antigen (CD70)	-	-	-	-	-	-	100	0.212	Yes [23]
Sushi-Repeat-Containing Protein, X Chromosome (SRPX)	-	-	-	-	-	-	100	0.179	-
Golgi Membrane Protein 1 (GOLM1)	100	0.995	-	-	58.319	0.995	100	0.166	-
Stanniocalcin 1 (STC1)	-	-	-	-	-	0.145	100	0.145	Yes [24]
Transmembrane Protein 59 (TMEM59)	-	-	-	-	-	-	100	0.129	-
Galactosylgalactosylxylosylprotein 3-Beta-Glucuronosyltransferase 2 (B3GAT2)	-	-	-	-	-	-	100	0.11	-
Isoform 3 of Tyrosine-Protein Kinase Lck (LCK)	-	-	-	-	-	-	100	0.08	Yes [25]
Chromosome 1 Open Reading Frame 112 (C1orf112)	1.368	0.061	-	-	-	-	100	0.061	-
Isoform 2 of Alpha,6-Mannosylglycoprotein 6-Beta-N-Acetylglucosaminyltransferase B (MGAT5B)	-	-	-	-	-	-	100	0.047	-
Laminin Subunit Beta 2 (LAMB2)	100	0.021	-	-	51.099	0.043	34.822	0.065	-
Transferrin (TF)	-	-	-	-	-	-	33.341	0.166	Yes [26]
Carboxypeptidase (CTSA)	3.04	0.096	1.641	0.905	2.4865	0.318	21.021	0.738	Yes [3]
Fibrinogen-Like Protein 1 (FGL1)	-	-	-	-	-	-	14.448	0.136	-
Tyrosine-Protein Kinase Receptor (SDC4-ROS1_S4;R32)	-	-	-	-	100	0.061	7.664	0.061	-
Fructose-Bisphosphate Aldolase (ALDOC)	-	-	0.542	1.593	-	-	6.13	4.736	Yes [27]
Insulin-Like Growth Factor-Binding Protein 3 (IGFBP3)	-	-	-	-	37.926	0.931	6.039	0.551	Yes [28]
MucinB (MUC5B)	-	-	-	-	-	-	3.878	0.746	-
Pleckstrin Homology Domain-Containing Family B Member 2 (PLEKHB2)	-	-	-	-	-	-	3.853	0.116	-
Eukaryotic Initiation Factor 4A-III (EIF4A3)	1.6715	13.454	-	-	1.5165	19.893	2.378	0.318	-
Hepatocyte Growth Factor Receptor (MET)	13.464	0.415	-	-	1.06	0.658	2.265	0.032	Yes [29]
Isoform 2 Procollagen-Lysine,2-Oxoglutarate 5-Dioxygenase 2 (PLOD2)	1.9435	3.885	0.586	0.848	10.655	7.149	2.227	3.642	Yes [30]
Ferritin Heavy Chain (FTH1)	-	0.359	1.16	12.594	8.775	0.166	2.177	20.544	Yes [31]

**Table 2:** Membrane protein components of A549 lung-cancer-derived EVs.

Integral Membrane Proteins						Plasma Membrane Proteins						
Nx EV Gene Symbols	H/L	emPAI	Hx EV Gene Symbols	H/L	emPAI		Nx EV Gene Symbols	H/L	emPAI	Hx EV Gene Symbols	H/L	emPAI
***CD9***	0.531	26.826	***CD9***	0.368	11.915		***CTSD***	11.052	366.47	***CTSD***	6.785	271.13
***CD151***	0.415	0.179	***CD151***	0.269	0.179		***GNAS***	0.249	0.365	***GNAS***	0.168	0.283
***EPHA2***	0.152	0.172	***EPHA2***	0.020	0.22		***LIN7A***	0.161	0.179	***LIN7A***	0.172	0.179
***ATP1B1***	0.107	0.311	***ATP1B1***	0.095	0.311		***ARF4***	0.233	0.701	***ARF4***	0.173	0.701
***STX5***	0.201	0.585	***STX5***	0.113	0.259		***CAP1***	0.153	0.322	***CAP1***	0.259	1.009
***TSPAN9***	0.994	0.425	***TSPAN9***	0.663	0.194		***RAB5C***	0.321	1.054	***RAB5C***	0.205	1.738
***CAV1***	0.158	0.259	***CAV1***	0.180	0.585		***RAB34***	0.164	0.413	***RAB34***	0.194	0.259
***QSOX1***	46.856	0.501	***QSOX1***	15.013	1.581		***RAB21***	0.265	0.166	***RAB21***	0.097	0.166
***ITGB1***	0.126	1.239	***ITGB1***	0.088	3.217		***RAB10***	0.130	1.254	***RAB10***	0.225	1.955
***TSPAN15***	0.284	0.389	***TSPAN15***	0.101	0.179		***SDCBP***	2.380	128	***SDCBP***	1.567	17.957
***NUTF2***	0.010	0.334	***NUTF2***	0.010	0.334		***RAB14***	0.010	0.585	***RAB14***	0.167	0.848
***PLXNB2***	0.010	0.022	***PLXNB2***	0.025	0.067		***EFNB1***	1.149	0.11	***EFNB1***	0.647	0.233
***CD81***	0.950	6.943	***CD81***	0.648	5.31		***RHOG***	0.160	0.468	***RHOG***	0.040	0.212
***SLC16A1***	0.135	0.311	***SLC16A1***	0.061	0.501		***GNAI2***	0.010	0.389	***GNAI2***	0.010	0.551
***GLUD1***	0.092	0.896	***GLUD1***	0.068	0.896		***RAB2A***	0.155	1.929	***RAB2A***	0.258	0.585
***AXL***	3.440	0.179	***AXL***	2.364	0.245		***DSC1***	0.010	0.094	***DSC1***	0.010	0.094
***QSOX2***	0.230	0.222	***QSOX2***	0.073	0.284		***RHOB***	1.273	0.52	***RHOB***	1.861	0.52
***TSPAN14***	0.850	4.179	***TSPAN14***	0.661	2.728		***CP***	19.204	0.52	***CP***	11.341	7.111
***NTRK3***	1.113	0.116	***NTRK3***	0.548	0.116		***RAB7A***	0.209	1.254	***RAB7A***	0.175	1.254
**-**	-	-	***PLEKHB2***	3.853	0.116		***GNA13***	0.397	0.318	***GNA13***	0.010	0.318
**-**	-	-	***TPR***	0.010	0.016		***DSG1***	0.010	0.054	***DSG1***	0.010	0.054
**-**	-	-	***MET***	2.265	0.032		***DMBT1***	0.010	0.056	***DMBT1***	0.010	0.056
**-**	-	-	***EGFR***	0.448	0.033		***JUP***	0.010	0.17	***JUP***	0.010	0.17
**-**	-	-	***PTGFRN***	0.373	0.136		***CD44***	0.186	0.968	***CD44***	0.084	0.719
							***GNAI3***	0.010	0.438	***GNAI3***	0.030	0.624
							***RAB32***	0.344	0.155	***RAB32***	0.361	0.155
							***RAB11A***	0.244	0.501	-	-	-
							***TTYH3***	100	0.11	-	-	-
							***CDH1***	2.675	0.194	-	-	-
							***PLSCR1***	0.637	0.52	-	-	-
							***RHOC***	0.532	0.468	-	-	-
							***SNAP23***	0.010	0.179	-	-	-
							***CDC42***	0.254	0.52	-	-	-
							***RAB8B***	0.387	1.054	-	-	-
							-	-	-	***RAB11B***	0.212	0.778
							-	-	-	***CLTB***	0.010	0.259
							-	-	-	***CDH2***	0.331	0.061
							-	-	-	***RAB31***	0.010	0.179
							-	-	-	***GNAI1***	0.118	0.833
							-	-	-	***IGHG1***	0.010	0.105
							-	-	-	***PKP1***	0.105	0.043
							-	-	-	***CD70***	100	0.212
							-	-	-	***U3KQV3***	0.437	0.166

**Table 3:** EV proteins involved in intracellular membrane trafficking.

Nx EV Gene Symbol	H/L	emPAI	Hx EV Gene Symbol	H/L	emPAI
***VPS35***	0.254	0.047	***VPS35***	0.140	0.259
***STX5***	0.201	0.585	***STX5***	0.113	0.259
***SDCBP***	2.380	128	***SDCBP***	1.567	17.957
***CAV1***	0.158	0.259	***CAV1***	0.180	0.585
***VPS29***	0.490	0.778	***VPS29***	0.261	1.61
***LMAN2***	0.886	0.931	***LMAN2***	0.403	0.551
***VPS26A***	0.370	0.233	***VPS26A***	0.135	0.369
***IGF2R***	0.444	0.418	***IGF2R***	0.344	1.284
***PDCD6IP***	0.305	0.874	***PDCD6IP***	0.285	1.125
***CLTC***	0.441	1.976	***CLTC***	0.183	4.867
***SNAP23***	0.010	0.179	***-***	-	-
***SYNGR2***	0.010	0.292	***-***	-	-
**-**	-	-	***CLTB***	0.010	0.259
**-**	-	-	***GOSR1***	0.254	0.122
**-**	-	-	***COPA***	0.010	0.124
**-**	-	-	***COPE***	0.126	0.116
**-**	-	-	***COPB1***	0.010	0.041
**-**	-	-	***COPG1***	0.010	0.045

## Supplementary Materials

The following are available online at https://www.mdpi.com/2072-6694/12/10/2917/s1, Figure S1: Detailed information about western blot, Figure S2: Validation of newly synthesized proteins using Western blots, Figure S3: Annotation clusters of extracellular matrix related protein found in EVs derived from A549 cells cultured under normal culture conditions, Figure S4: Annotation clusters of extracellular matrix related protein found in EVs derived from A549 cells cultured under hypoxia (Hx), Figure S5: Gene ontology analysis revealed biological process associated with proteins (n = 443) found in both condition (Nx and Hx) that had similar H/L ratios, Table S1: List of top 25 most commonly identified EV markers from Exocarta, Table S2: List of top 25 most commonly identified EV markers from Vesiclepedia, File S1: A549 EVs pSILAC, File S2: A549 cell lysate pSILAC.
