# Supplementary material for: Microenvironmental Hypoxia Induces Dynamic Changes in Lung Cancer Synthesis and Secretion of Extracellular Vesicles
Source: Cancers (Basel). 2020 Oct 11;12(10):2917. doi: 10.3390/cancers12102917 (PMC7601203; doi:10.3390/cancers12102917)
Supplement: Supplementary file 1 [file cancers-12-02917-s001.zip › Supplementary Figures.pdf]

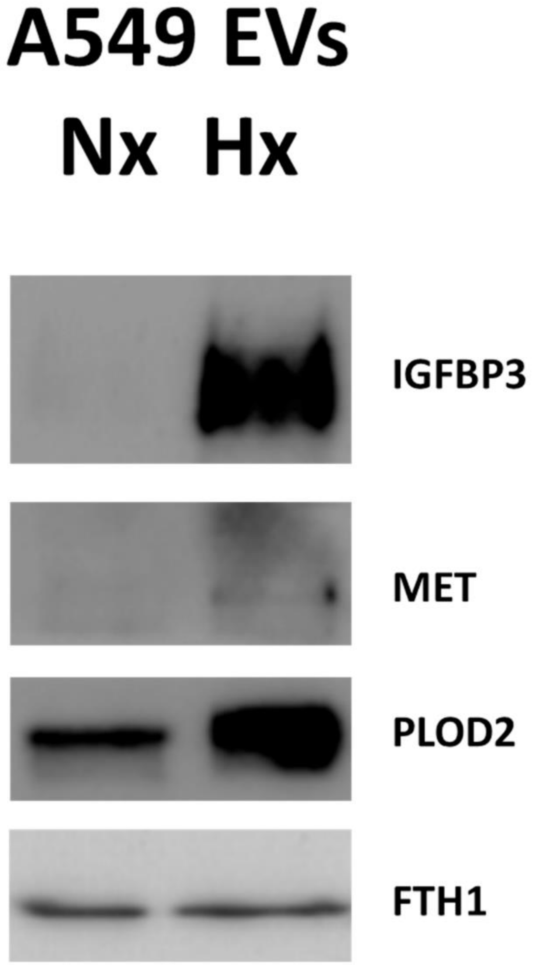

Figure S1. Validation of newly synthesized proteins using Western blots. Several EVs proteins that were classified as newly synthesised in Table 1 were selected and validated using non-SILAC labelled EVs isolated from A549 lung cancer cells cultured under normal culture conditions or hypoxia. Experiment was repeated with 3 biological replicate.

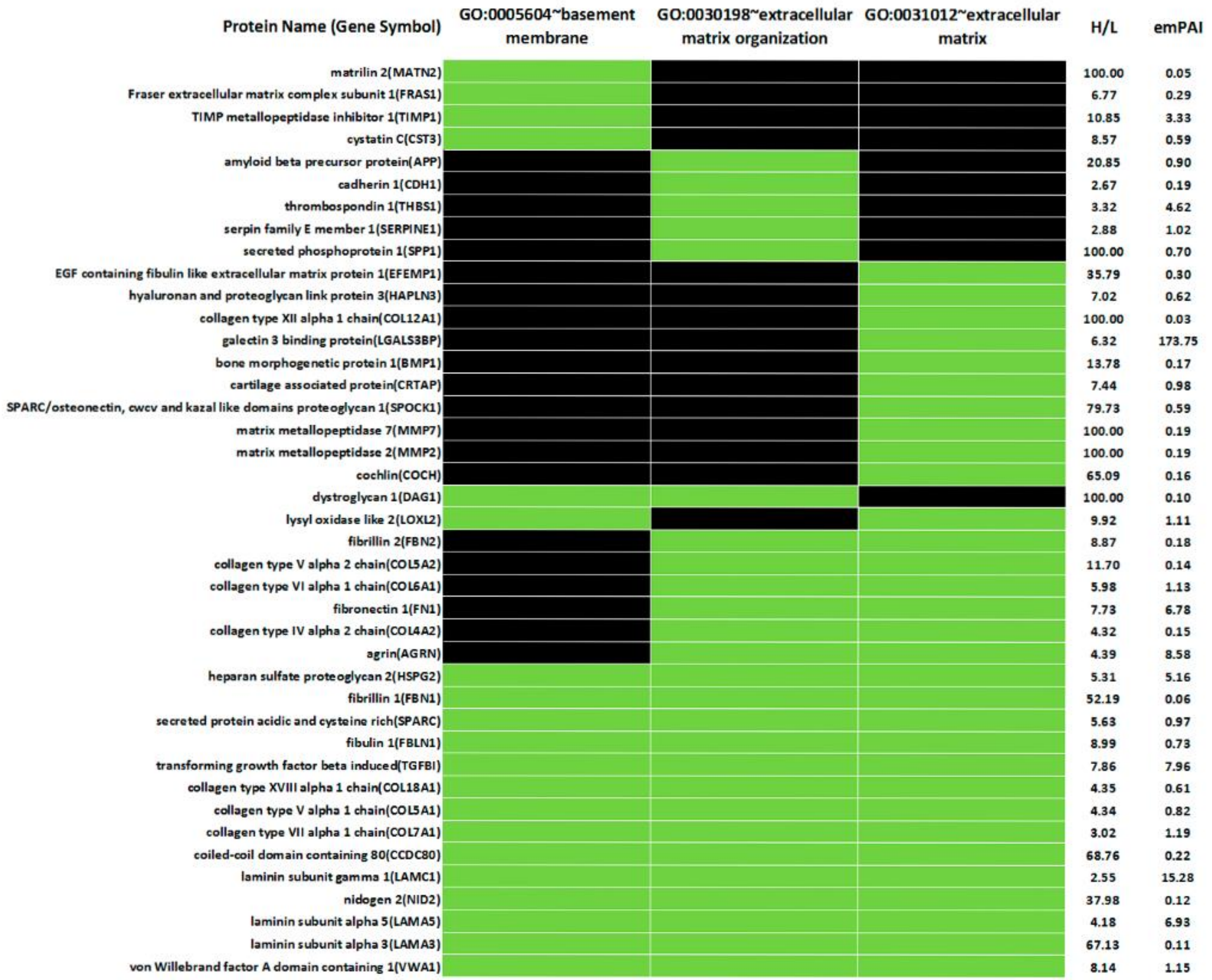

Figure S2. Annotation clusters of extracellular matrix related protein found in EVs derived from A549 cells cultured under normal culture conditions. EV proteins associated with the corresponding gene terms are shown in green. Proteins not reported to be associated with the gene terms are shown in black. H/L ratios and relative abundance (emPAI) of each proteins were extracted from pSILAC data.

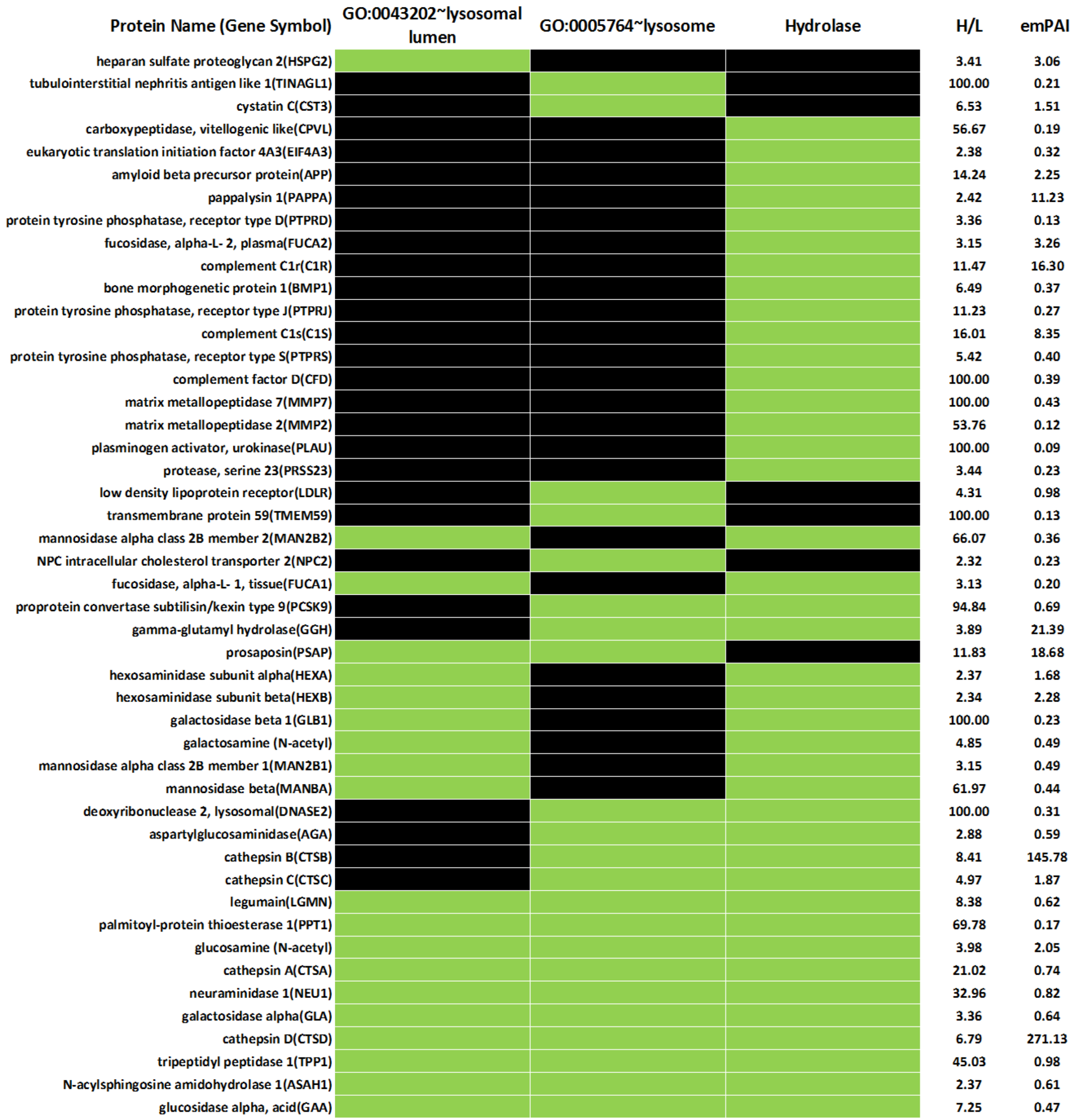

Figure S3. Annotation clusters of extracellular matrix related protein found in EVs derived from A549 cells cultured under hypoxia (Hx). EV proteins associated with the corresponding gene terms are shown in green. Proteins not reported to be associated with the gene terms are shown in black. H/L ratios and relative abundance (emPAI) of each proteins were extracted from pSILAC data.

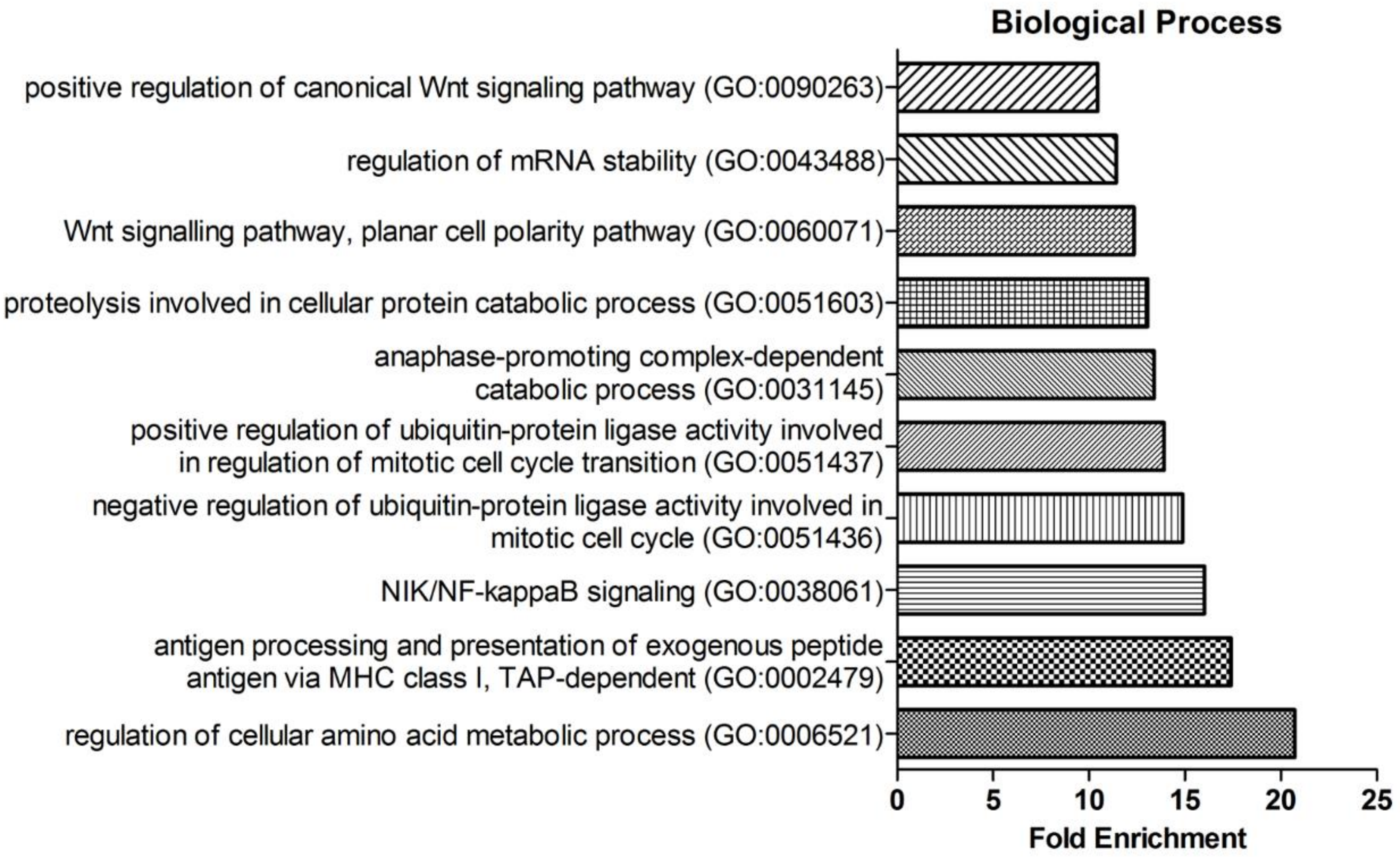

Figure S4. Gene ontology analysis revealed biological process associated with proteins (n=443) found in both condition (Nx and Hx) that had similar H/L ratios.

Supplementary Figure 5: Uncropped western blot images

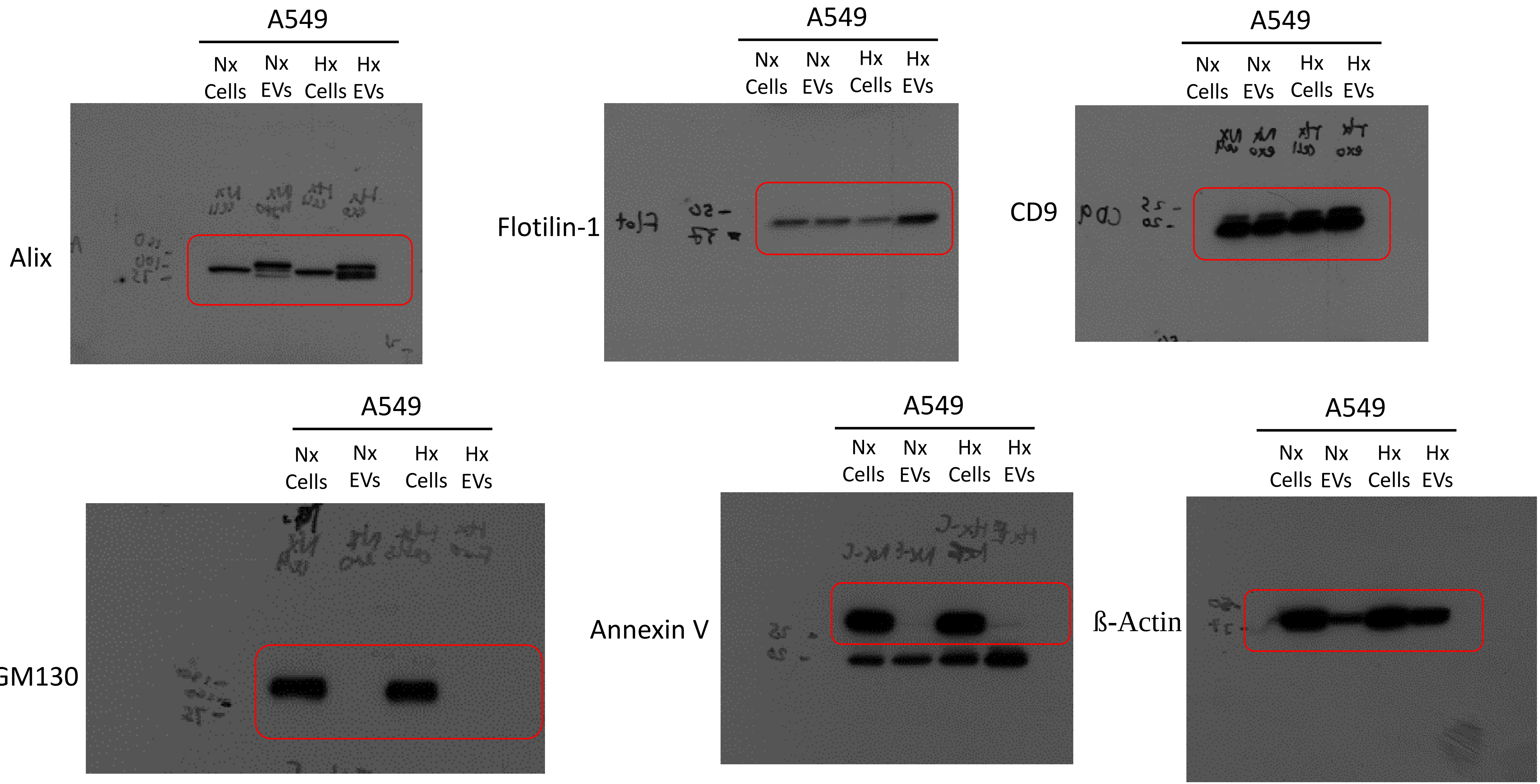

Uncropped western blot images for Figure 1D

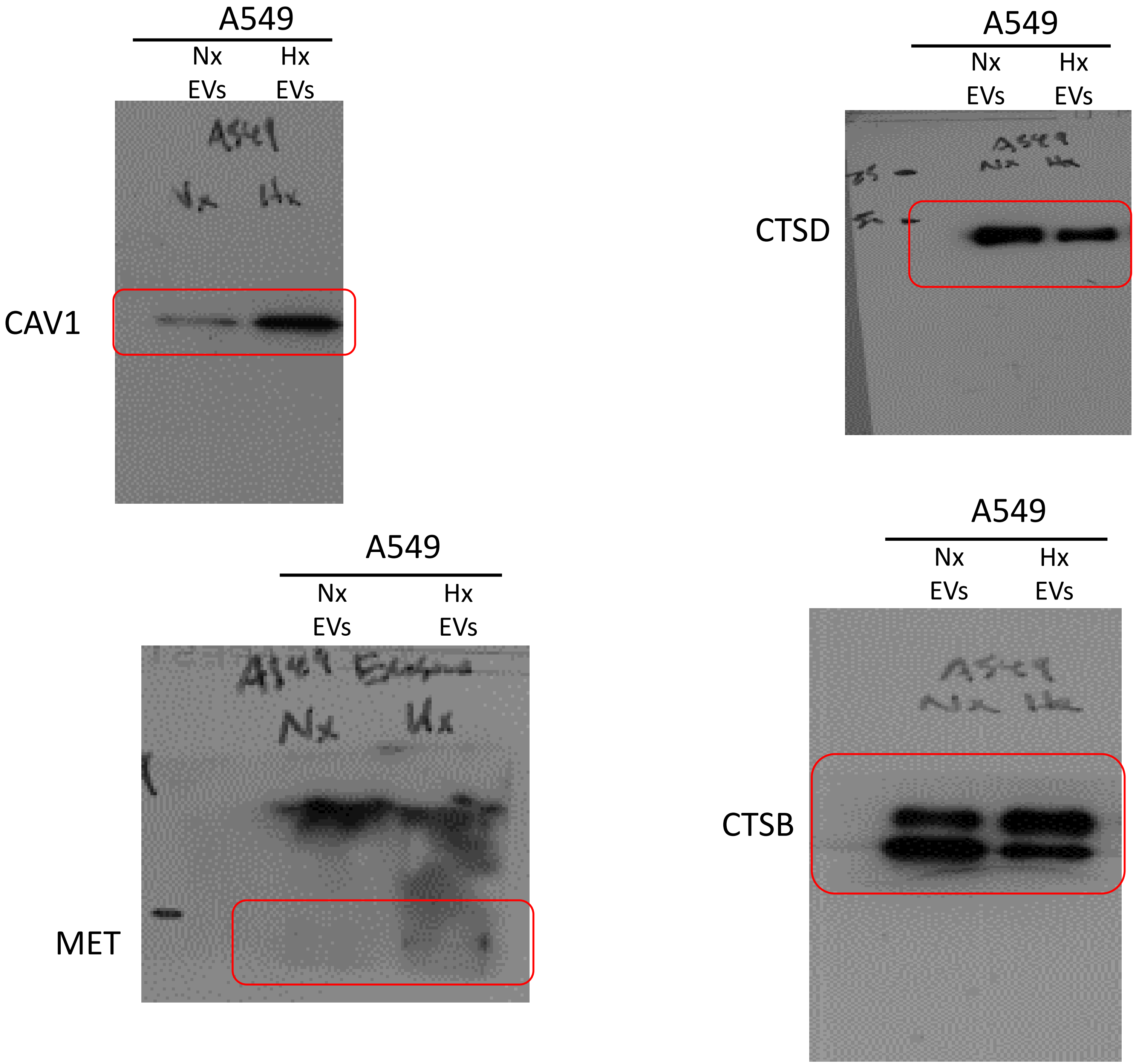

Uncropped western blot images for Figure 3B

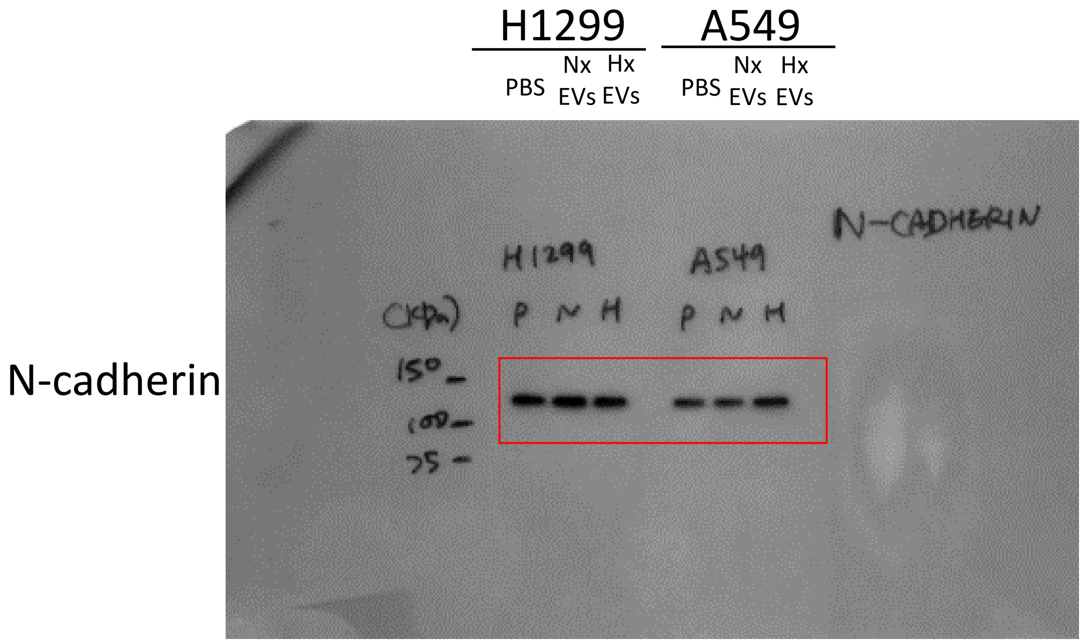

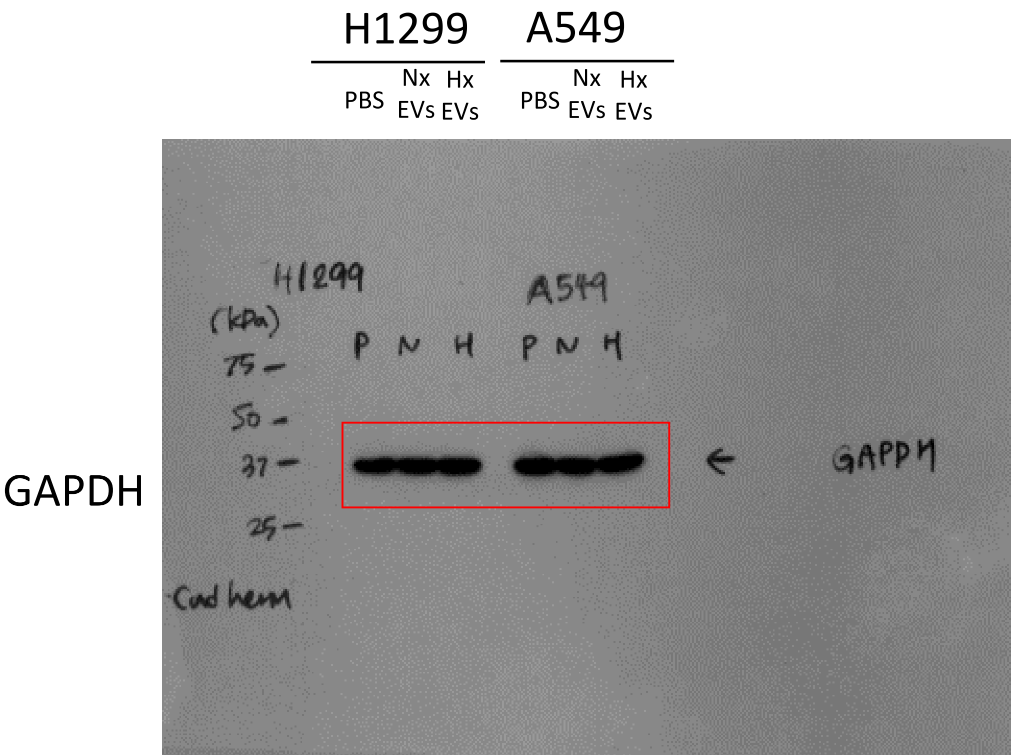

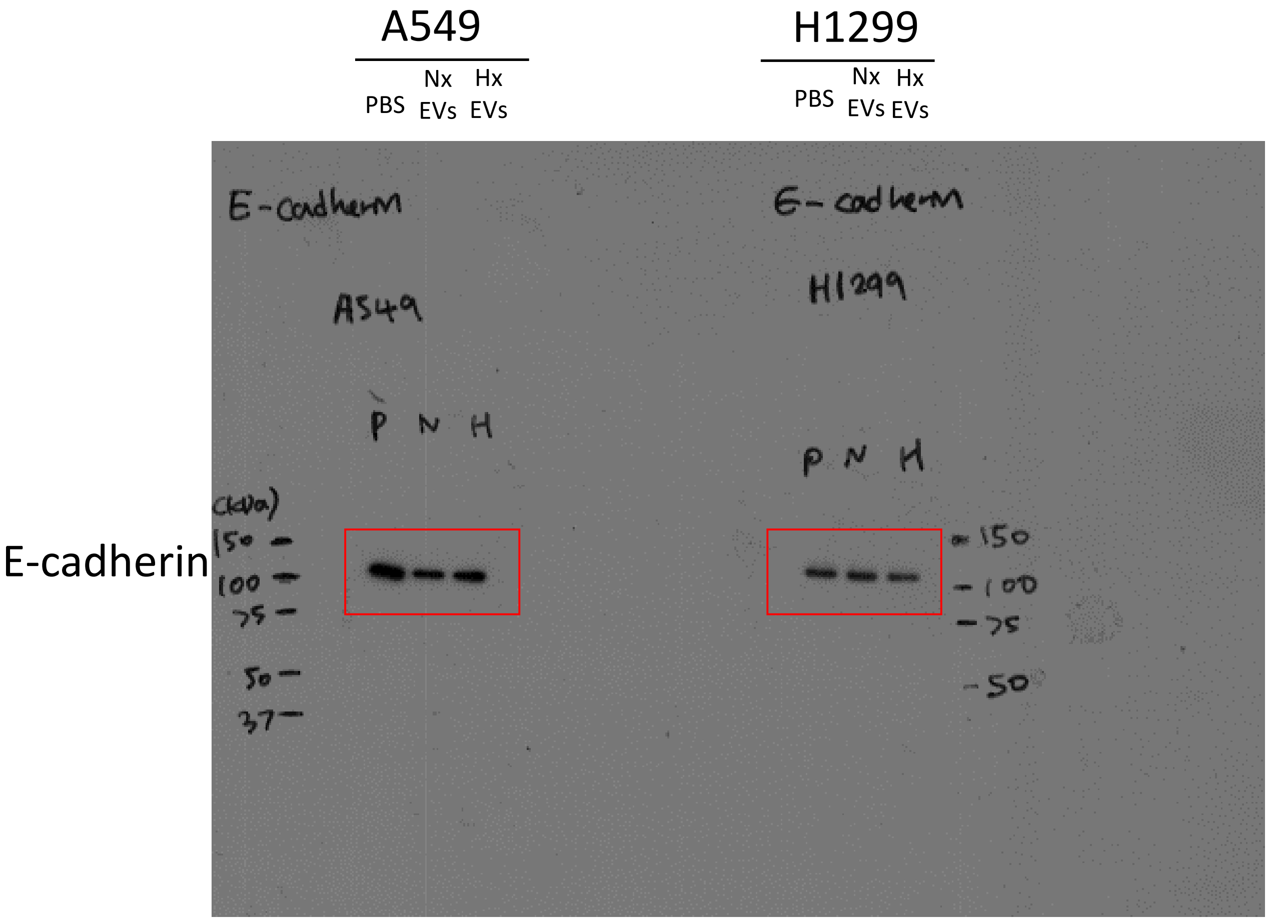

Uncropped western blot image of Figure 4C

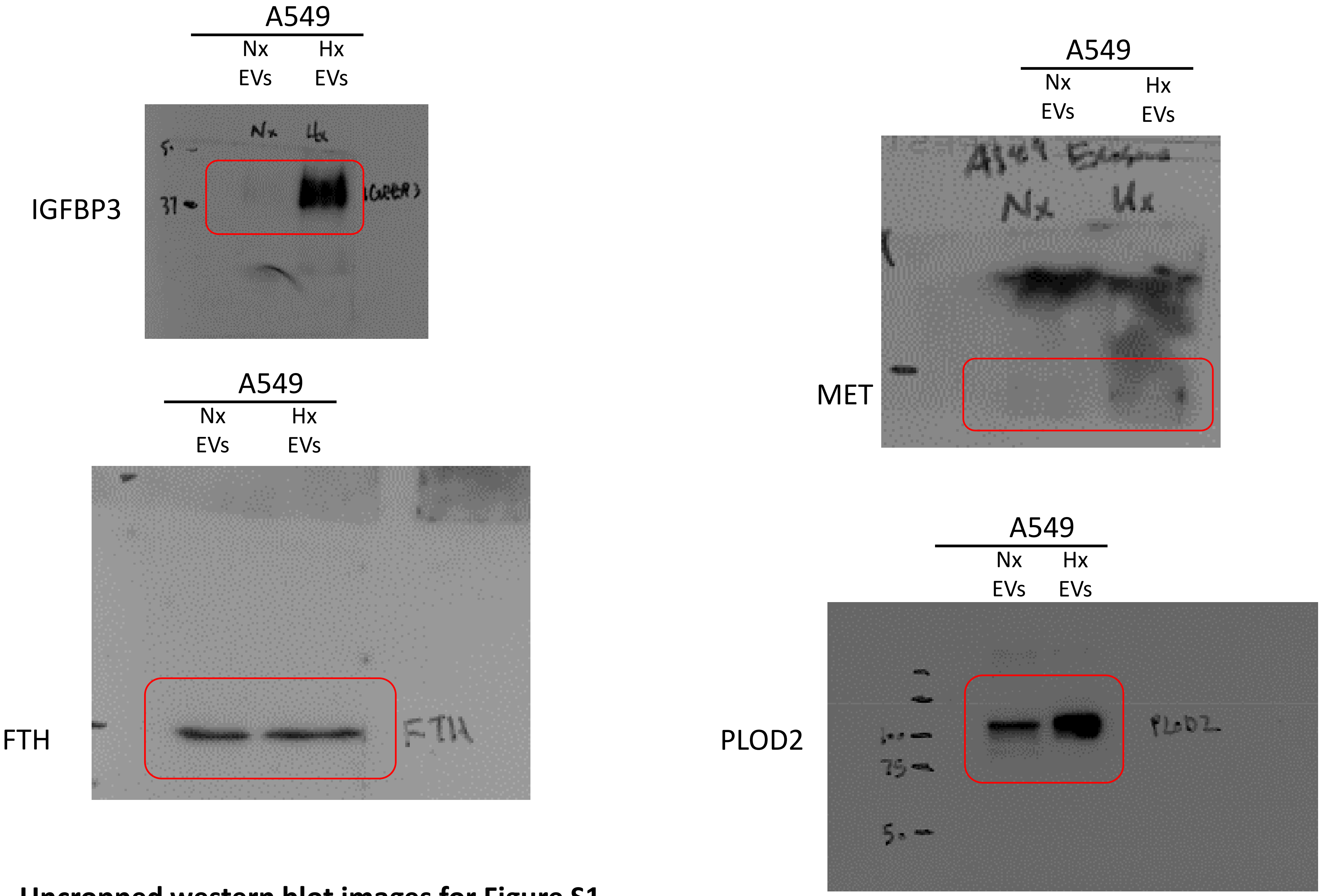

Uncropped western blot images for Figure S1
