# Supplementary material for: Microenvironmental Hypoxia Induces Dynamic Changes in Lung Cancer Synthesis and Secretion of Extracellular Vesicles
Source: Cancers (Basel). 2020 Oct 11;12(10):2917. doi: 10.3390/cancers12102917 (PMC7601203; doi:10.3390/cancers12102917)
Supplement: Supplementary file 1 [file cancers-12-02917-s001.zip › Supplementary Table.pdf]

Table S.1

List of top 25 most commonly identified EV markers from Exocarta

Gene Symbol	NxEVs H/L	NxEVs emPAI	HxEVs H/L	HxEVs emPAI
**HSPA8**	0.16	2.07	0.04	2.89
**CD9**	0.53	26.83	0.37	11.92
**GAPDH**	0.26	12.90	0.14	172.02
**ACTB**	0.22	271.13	0.13	221.75
**CD63**	N/A	N/A	N/A	N/A
**CD81**	0.95	6.94	0.65	5.21
**ANXA2**	0.15	120.15	0.16	463.16
**ENO1**	0.24	19.43	0.23	136.38
**HSP90AA1**	0.16	4.18	0.09	9.48
**EEF1A1**	0.09	2.31	0.02	2.02
**PKM**	0.19	2.93	0.24	7.30
**YWHAE**	0.20	76.43	0.15	39.84
**SDCBP**	2.38	128.16	1.57	17.96
**PDCD6IP**	0.13	2.62	0.07	4.26
**ALB**	0.01	2.94	0.01	3.64
**YWHAZ**	0.23	25.37	0.21	5.95
**EEF2**	0.01	0.09	0.01	0.41
**ACTG1**	N/A	N/A	N/A	N/A
**LDHA**	0.17	38.81	0.20	108.65
**HSP90AB1**	0.13	2.62	0.07	4.26
**ALDOA**	0.27	80.86	0.36	331.46
**MSN**	0.09	0.32	0.08	1.08
**ANXA5**	0.25	1.65	0.18	1.22
**PGK1**	0.17	0.85	0.77	4.41
**CFL1**	0.19	2.16	0.15	2.16

Table S.2

List of top 25 most commonly identified EV markers from Vesiclepedia

Gene Symbol	NxEVs H/L	NxEVs emPAI	HxEVs H/L	HxEVs emPAI
**PDCD6IP**	0.13	2.62	0.07	4.26
**GAPDH**	0.26	12.90	0.14	172.02
**HSPA8**	0.16	2.07	0.04	2.89
**ACTB**	0.22	271.13	0.13	221.75
**ANXA2**	0.15	120.15	0.16	463.16
**CD9**	0.53	26.83	0.37	11.92
**PKM**	0.19	2.93	0.24	7.30
**HSP90AA1**	0.16	4.18	0.09	9.48
**ENO1**	0.24	19.43	0.23	136.38
**ANXA5**	0.25	1.65	0.18	1.22
**HSP90AB1**	0.13	2.62	0.07	4.26
**CD63**	N/A	N/A	N/A	N/A
**YWHAZ**	0.23	25.37	0.21	5.95
**YWHAE**	0.20	76.43	0.15	39.84
**EEF1A1**	0.09	2.31	0.02	2.02
**PGK1**	0.17	0.85	0.77	4.41
**CLTC**	0.44	1.98	0.18	4.87
**PPIA**	0.20	0.59	0.11	1.15
**SDCBP**	2.38	128.16	1.57	17.96
**ALDOA**	0.27	80.86	0.36	331.46
**EEF2**	0.01	0.09	0.01	0.41
**ALB**	0.01	2.94	0.01	3.64
**TPI1**	0.18	28.76	0.27	36.93
**VCP**	0.20	27.73	0.14	52.60
**CFL1**	0.19	2.16	0.15	2.16
